# Updated cost-effectiveness analysis of lung cancer screening for Australia, capturing differences in the health economic impact of NELSON and NLST outcomes

**DOI:** 10.1038/s41416-022-02026-8

**Published:** 2022-11-03

**Authors:** Silvia Behar Harpaz, Marianne F. Weber, Stephen Wade, Preston J. Ngo, Pavla Vaneckova, Peter E. A. Sarich, Sonya Cressman, Martin C. Tammemagi, Kwun Fong, Henry Marshall, Annette McWilliams, John R. Zalcberg, Michael Caruana, Karen Canfell

**Affiliations:** 1 The Daffodil Centre, the University of Sydney, A joint venture with Cancer Council NSW, Sydney, NSW Australia; 2 Faculty of Health Sciences, Simon Fraser University, Vancouver, BC Canada; 3 Department of Health Sciences, Brock University, St Catharines, ON Canada; 4 Department of Thoracic Medicine, The Prince Charles Hospital, Chermside, QLD Australia; 5 University of Queensland Thoracic Research Centre at The Prince Charles Hospital, Chermside, QLD Australia; 6 Fiona Stanley Hospital, Murdoch, WA Australia; 7 School of Public Health and Preventive Medicine, Monash University, Melbourne, VIC Australia

**Keywords:** Population screening, Health policy

## Abstract

**Background:**

A national, lung cancer screening programme is under consideration in Australia, and we assessed cost-effectiveness using updated data and assumptions.

**Methods:**

We estimated the cost-effectiveness of lung screening by applying screening parameters and outcomes from either the National Lung Screening Trial (NLST) or the NEderlands–Leuvens Longkanker Screenings ONderzoek (NELSON) to Australian data on lung cancer risk, mortality, health-system costs, and smoking trends using a deterministic, multi-cohort model. Incremental cost-effectiveness ratios (ICERs) were calculated for a lifetime horizon.

**Results:**

The ICER for lung screening compared to usual care in the NELSON-based scenario was AU$39,250 (95% CI $18,150–108,300) per quality-adjusted life year (QALY); lower than the NLST-based estimate (ICER = $76,300, 95% CI $41,750–236,500). In probabilistic sensitivity analyses, lung screening was cost-effective in 15%/60% of NELSON-like simulations, assuming a willingness-to-pay threshold of $30,000/$50,000 per QALY, respectively, compared to 0.5%/6.7% for the NLST. ICERs were most sensitive to assumptions regarding the screening-related lung cancer mortality benefit and duration of benefit over time. The cost of screening had a larger impact on ICERs than the cost of treatment, even after quadrupling the 2006–2016 healthcare costs of stage IV lung cancer.

**Discussion:**

Lung screening could be cost-effective in Australia, contingent on translating trial-like lung cancer mortality benefits to the clinic.

## Introduction

Lung cancer is the leading cause of cancer-related death, both in Australia and worldwide [1]. Poor prognosis, poor quality of life and substantial healthcare resource requirements mean there is a continuing need for effective and cost-effective lung cancer control interventions. Primary prevention through tobacco control is likely to remain the most effective, equitable, and cost-effective long-term strategy for reducing the burden of lung cancer. However, given the 20–30 year lag between population-level tobacco exposure and lung cancer incidence, the full benefits of these interventions will not be realised for many years to come [2]. A population-based lung cancer screening programme has the potential to mitigate the adverse impact of historical smoking trends on health, and save many lives by detecting lung cancer at an early stage.

Two randomised controlled trials have demonstrated a significant lung cancer mortality reduction among individuals with a history of heavy tobacco exposure screened with low-dose computed tomography (LDCT). The U.S. National Lung Screening Trial (NLST) in 2011 [3] found a 20% (95% confidence interval; CI 6.8%–26.7%) lung cancer mortality reduction and more recently, the NEderlands–Leuvens Longkanker Screenings ONderzoek (NELSON) [4] demonstrated a 24% (cumulative rate ratio 0.76, 95% CI 0.61–0.94) reduction for men (33% for women; cumulative rate ratio 0.67, 95% CI 0.38–1.14). These trials resulted in several agencies recommending lung cancer screening for people at high risk, including the U.S. Preventative Services Task Force (USPSTF [5, 6]), and health economic evaluations of lung screening have demonstrated favourable cost-effectiveness estimates in many settings [7–11].

In 2021, an Australian Government-led enquiry recommended the implementation of a population-wide, risk-based lung screening programme [12]. Previous published health economic evaluations of lung cancer screening in the Australian setting found that LDCT screening was not likely to be cost-effective [13, 14], however, the evidence on lung screening has advanced since those reports, including the results of the NELSON trial. We provide an updated cost-effectiveness estimate for the Australian healthcare system by using new modelled data on Australian rates of smoking initiation and cessation, Australian lung cancer mortality and survival, as well as updated Australian health services costs, to assess the economic impact given the lung cancer screening outcomes observed in the NELSON and NLST trials.

## Methods

**Fig. 1:**
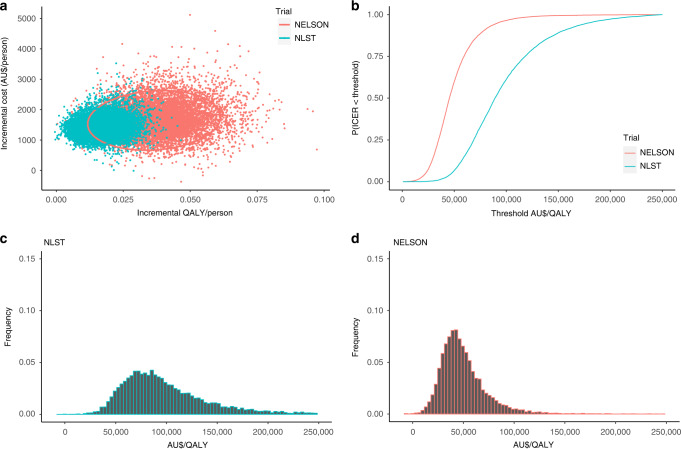
Probabilistic sensitivity analysis of lung cancer screening cost-effectiveness, given each trial setting in the Australian population. **a** Scatter plot of incremental costs (in AU$/person) vs incremental QALYs/person obtained from the PSA for the NELSON and NLST settings. **b** Corresponding estimated cost-effectiveness curve given the ICER distributions obtained from the PSA. **c**, **d** Histograms showing the ICER distributions obtained from the PSA for the NLST and NELSON settings, respectively. ICER Incremental cost-effectiveness ratio, QALY quality-adjusted life-year, NELSON Nederlands–Leuvens Longkanker Screenings Onderzoek, NLST National Lung Screen Trial, PSA probabilistic sensitivity analysis.

A cumulative lifetime risk model was implemented in R [15, 16], where Australian rates of all-cause and lung cancer (LC) mortality by sex, age and smoking status were used to estimate the number of LC cases and life-years/quality-adjusted life-years (LYs/QALYs) gained in a hypothetical scenario comparing a screened (applying NLST or NELSON trial parameters) versus unscreened population. That is, we modelled the direct impact of the screening-related lung cancer mortality benefit observed in the trials on population-level lung cancer mortality rates. Incremental costs, incremental benefits and the incremental cost-effectiveness ratio (ICER) of each trial setting in the Australian population compared to usual care were estimated. A probabilistic sensitivity analysis was used to determine the 95% confidence intervals for incremental costs, benefits, and ICERs for our base case (see Fig. 1a). A universal public payer perspective was taken. Detailed information on the model, data inputs, parameters and assumptions are provided in the Supplementary Appendix.

### Data inputs

#### Screening parameters

Data on the eligible age range, the number of screens, screening-related LC stage shift, follow-up CT rate, false-positive rate, overdiagnosis, and LC mortality benefit were ascertained from the NLST and the NELSON trial and modelled separately according to each trial (Table 1) [3, 4, 17]. For the NLST, false positive scans were partitioned into those requiring a follow-up CT scan (~15% of scans) and those requiring other diagnostic work-up (~8.5%) [3].

**Table 1:** Model specifications, parameters, structural assumptions, and base-case values.

Specification	Key assumptions and sources	Base-case values			
Model type	Deterministic, multi-cohort, trial setting				
Target population	Individuals with a history of heavy tobacco smoking in Australia selected according to the NLST or NELSON eligibility criteria.				
Intervention	LDCT screening				
Comparator	No screening (usual care)				
Willingness- to- pay threshold	$30,000–$50,000/ QALYS, consistent with previous Medical Services Advisory Committee evaluations.				
Currency	2021 Australian Dollars				
		**NLST**		**NELSON**	
Eligible participants	Trial-based criteria [3, 4] applied to the 45 and Study cohort [21, 22]	Current Smoking	36.1%	Current Smoking	50.1%
		Former Smoking	63.9%	Former Smoking	49.9%
		Age		50–54	29.6%
		55–59	34.6%	55–59	27.7%
		60–64	31.4%	60–64	21.3%
		65–69	22.1%	65–69	14.6%
		70–74	12.0%	70–74	6.8%
				75–80	0.0%
		Men	60.8%	Men	55.0%
		Women	39.2%	Women	45.0%
LC screening mortality benefit	Trial-based [3, 4, 17]. Constant LC mortality benefit during the screening phase	0.2		0.24	
Mortality benefit after trial ends	Trial-based [3, 4, 17]	Decreases linearly to 0 in 3 years		Decreases linearly to 0 in 4 years	
Follow-up CT rate	Trial-based [3, 4, 17]	0.151		0.1	
False-positive rate	Trial-based [3, 4, 17]	0.082		0.012	
Overdiagnosis (excess LC incidence in the LDCT arm out of all LC cases in the LDCT arm)	Trial-based [3, 4, 17]	1.9%		5.3%	
Screen time points	Trial-based [3, 4, 17]	0, 1, 2		0, 1, 3, 5	
LC death HR by eligibility criteria	Relative to never-smoking, from the 45 and Up Study [24]	HR = 30.12 (current); HR = 16.91 (former)		HR = 26.87 (current); HR = 18.47 (former)	
All-cause mortality HR	Relative to never-smoking [18]	HR by sex and age group (given in the Supplementary appendix)			
Time horizon		Lifetime			
LC survival data	National rates from AIHW [23]. The number of LC cases was estimated from the number of LC deaths by applying 5-year survival.	5-year survival			
Cost of LDCT scan	Australian MBS fee	$302			
Average cost of follow-up CT	QLCSS [26]	$476			
Cost false positive	QLCSS [26]. Non-CT work-up	$861.8			
Costs of LC management by stage	Health-system costs based on the 45 and up study [25] (inclusive of pre-diagnosis costs)	Stage I: $62,327; Stage II: $69,235; Stage III: $63,436; Stage IV: $56,701; Stage “unknown”: $38,388			
Cost pre-diagnosis	Health-system costs based on the 45 and up study [25]. The same cost applied to screen and no-screen scenarios.	Stage I: $6,010; Stage II: $6,010; Stage III: $4,196; Stage IV $2,427; Stage “unknown”: $4,606			
Cases in stage “unknown” (estimated)		0 cases in the screening scenario· “Unknown” composition: 60% IV, 21% III, 12% II, 7% I			
Stage shift	Trial-based [3, 4, 17] survival rates and stage distribution at diagnosis were independent of smoking status. Survival rates were assumed equivalent for screened and unscreened cases.	Based on NLST trial data		Based on NELSON trial data	
Baseline utility weights	From the 45 and Up Study. A drop in the baseline and LC utilities of 0.01 was assumed after age 70 and of 0.04 after age 80 [28].	0.787 (men); 0.749 (women)		0.779 (men); 0.757 (women)	
Screening disutility		Screening: 0.02 (2 weeks); follow-up CT: 0.02 (3 months); false-positive scan: same as stage I LC (3 months)			
LC utility weights	Based on the Cancer Care Outcomes Research and Surveillance Study [34]. LC Utility weights were applied for the remainder of the survival period.	Stage I: 0.71; Stage II: 0.68; Stage III: 0.67; Stage IV: 0.66; Stage “unknown”: 0.68. A drop in utilities of 0.01 was assumed after age 70 and of 0.04 after age 80.			
Discount rate		5% on costs and benefits			

*AIHW* Australian Institute of Health and Welfare, *HR* hazard ratio, *LC* lung cancer, *LDCT* low-dose computed tomography, *LYs* Life-years, *MBS* Medicare Benefits Schedule, *NELSON* Nederlands–Leuvens Longkanker Screenings Onderzoek, *NLST* National Lung Screen Trial, *QLCSS* Queensland Lung Cancer Screening Study.

#### Screening-eligible population

A compartmental model of smoking prevalence, detailed in Wade et al. [18], calibrated to the observed distribution of smoking status (current daily/never/former) among individuals aged 20–99 years from 1962–2016 by sex and birth year (1910–1996) [19], was used to estimate the prevalence of current and former smoking in Australia, 2020–2066. Estimates were simulated using 50-year population projections [20] and accounted for all-cause mortality rates stratified by smoking status [18]. In the base case, we applied the estimated background all-cause mortality rate of individuals whose smoking status was “current” to those with both “current” and “former” smoking status.

The proportion of Australians eligible for screening was estimated by applying the NLST and NELSON selection criteria to a population-based Australian cohort study, the Sax Institute’s *45 and Up Study* [21]. Detailed methods for ascertaining the proportion of screening-eligible participants in the cohort were published previously [22] and are described in the Supplementary Appendix. Ethical approval for the 45 and Up Study was provided by the University of NSW Human Research Ethics Committee and specific approval for this analysis was provided by the NSW Population and Health Services Research Ethics Committee. Informed consent was obtained from all participants.

#### Lung cancer incidence, mortality and stage distribution

The total number of LC cases diagnosed (excluding over-diagnosed cases) was assumed to be equal in the screening and no-screening scenarios and was obtained by dividing the number of LC deaths by the conditional probability of LC death given a LC diagnosis (which was approximated as one minus the 5-year relative LC survival by age and sex observed in Australia in 2011 [23]). For the screening scenario, the number of diagnosed cases was multiplied by a constant factor to account for overdiagnosis (5.3% of all LC cases in the screening arm for NELSON and 1.9% for NLST, see Supplementary Appendix).

Hazard ratios of LC mortality according to the NLST and NELSON eligibility criteria compared to ‘never-smoking’ status were derived from the *45 and Up Study*, using previously published methods [22, 24] and were used to estimate the number of LC deaths in each eligible sub-population by age, sex and smoking status (see Supplementary Appendix). The background LC mortality rate of ‘never-smoking’ status was calculated from LC mortality rates observed in Australia [23, 24] and the smoking prevalence obtained from our modelled estimates [18].

The Australian, age- and sex-specific distribution of LC cases by stage of disease at diagnosis was applied to cases in both scenarios, and then adjusted in the screening scenario by applying the stage shift observed in the trials; however, LC is often reported as “unknown stage” in Australia, thus these cases were re-classified as Stages I–IV based on observed survival rates (see Supplementary Appendix). Stage-specific LC relative survival was ascertained from the national database for cancers diagnosed 2012–2016 [23].

#### Costs

The average excess health-system costs of LC (compared to cancer-free controls) were estimated previously using the *45 and Up Study* for the years 2006–2016 [25]. Costs in the year prior to diagnosis and an initial treatment cost were applied to all cases. For cases who survived, an additional cost for the continuing care phase was applied, while for cases who died, a fraction of the continuing care cost (depending on the mean survival time by stage) and a terminal care phase cost were applied.

The calculated “average case” costs to 3 years by phase, were obtained by applying 1, 2 and 3-year survival data by stage of disease (see Supplementary Appendix). For the base case, the average costs were extended to 5 years (see Table 1). Note that in the screening scenario, the excess costs in the year prior to diagnosis were expected to be lower than usual care [25], given that screening eliminates the costs of diagnosing symptoms. Regardless, our base case assumed the same average pre-diagnosis costs in both scenarios.

The base-case cost of a LDCT scan was the price listed in the Medicare Benefits Schedule (AU$302 undiscounted in 2021), and the average cost of a follow-up CT and non-CT false-positive work-up were estimated from the Queensland Lung Cancer Screening Study [26]. All costs were presented in 2021 Australian dollars (1 AUD–0.72 USD) (Table 1). A constant discount rate of 5% was applied annually to all future costs and benefits from the beginning of the screening phase.

#### Utility weights

SF-6D utility values were derived from sources using the SF-12. Baseline utilities for screening-eligible men and women were derived from *the 45 and Up Study* [27] (Table 1). A drop in utility of 0.01 was applied at age 70–79, and 0.04 at age 80+ years [28].

Evidence of a measurable effect on quality of life following a positive or negative screening result is inconclusive [29–33]. Nevertheless, we conservatively applied a small, temporary disutility for LC screening itself (0.02 for 2 weeks). The same disutility was applied for 3 months to those that required follow-up CT. False-positive results requiring diagnostic work-up were assumed to incur the same utility as Stage I LC for 3 months.

Previously published LC utility weights [34] applied across the entire survival period, and decreased with age in the same way as individuals without LC (Table 1).

### Sensitivity analyses

One-way sensitivity analyses were undertaken to assess the single parameter uncertainty (see Supplementary Appendix), in which we varied assumptions regarding, the (1) screening mortality benefit observed in the trials; (2) relative risk of LC and all-cause mortality for individuals who were currently smoking or had quit smoking compared to those who never smoked; (3) costs of screening, LC diagnosis and treatment, and false positives, including a doubling and quadrupling of Stage IV total healthcare costs; (4) rate of false positives, follow-up CT scans, and overdiagnosis; (5) time horizon; (6) stage shift; (7) 5-year survival; (8) utility weights. Note that the mortality benefit is assumed to be an independent parameter in our model. Variations in the stage shift and 5-year survival had no influence on the mortality benefit (and thus on the LYs). The results of these variations should be interpreted as the effect that they have on the incremental cost and disutilities. The effect of stage shift on the LYs gained is indirectly captured by variations in the mortality benefit.

We also evaluated the effect of changing the eligibility criteria (i.e., changing the risk profile of the participants along with their LC mortality risk). We compared trial eligibility with selection criteria defined by the PLCO_m2012_ risk calculator [35] (PLCO_m2012_ ≥ 0.0151), and the USPSTF-2021 selection criteria [6]. The proportion eligible by age, sex, and smoking status was estimated from the 45 and Up Study cohort [22], and the LC death hazard ratio was estimated for sub-groups whose smoking status was ‘current’/‘former’- compared to ‘never’ for each selection criteria.

The combined parameter uncertainty throughout the model was evaluated with probabilistic sensitivity analysis (PSA) which simulated 10,000 possible ICER estimates by taking random values of all the model parameters (each independently) listed in Table 1 from each of the parameter distributions (except the time horizon and discount rate given that these are likely to be fixed, and the selection criteria) (see Supplementary Appendix).

We included an exploratory analysis investigating the effect of higher smoking cessation rates (by a factor of 1.2, 1.5 and 2) at every screening point among the participants in the screening scenario [36, 37], assuming a LC mortality benefit of 3% for each year since quitting [38, 39]. The last assumption underestimated the benefit of quitting since the all-cause mortality reduction was not included in this analysis. To maintain our conservative approach (i.e., less favourable for screening), we assumed the same (low) LC mortality benefit from quitting for both scenarios. The baseline cessation rates by age, sex and birth cohort were estimated using the Australian smoking behaviour model [18].

A threshold analysis on the cost of a LDCT screening scan was conducted using indicative willingness-to-pay thresholds of ~$30,000–$50,000 per QALY gained.

## Results

In the base case, the incremental number of LYs/QALYs per person, over a lifetime, was 0.038 (95% CI, 0.018–0.049)/0.019 (95% CI, 0.006–0.030) given the NLST trial and 0.067 (95% CI, 0.028–0.096)/0.041 (95% CI, 0.016–0.063) for the NELSON trial. The average incremental cost per person was $1434 (95% CI, $903–2097) for the NLST and $1606 (95% CI, $802–2762) for the NELSON trial, resulting in ICERs of $38,250 (95% CI, $24,400–83,550; NLST) and $24,050 (95% CI, $ 11,900–64,500; NELSON) per LY gained or $76,300 (95% CI, $41,750–236,500; NLST) and $39,250 (95% CI, $18,150–108,300; NELSON) per QALY gained.

The results of the PSA are presented in Fig. 1. The probability that the NELSON setting was cost-effective, assuming a willingness-to-pay threshold of $30,000 or $50,000/QALY gained was 15% and 60%, respectively, while the probability for the NLST was 0.5% and 6.7%.

**Fig. 2:**
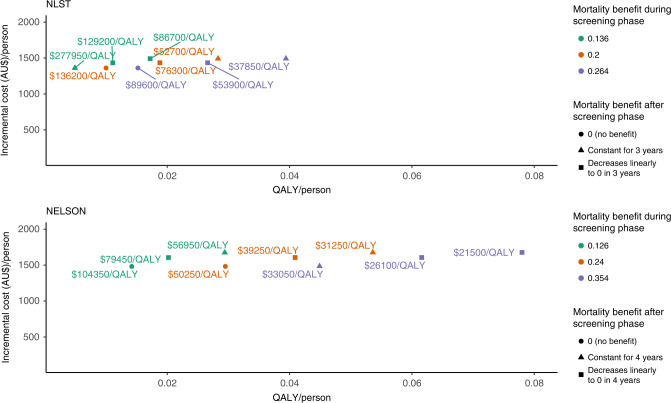
Incremental cost (in AU$/person) vs incremental QALYs/person for the NLST and NELSON settings, obtained by varying the assumptions related to the mortality benefit. QALY quality-adjusted life-year, NELSON Nederlands–Leuvens Longkanker Screenings Onderzoek, NLST National Lung Screen Trial.

Of all the model parameters, variations in the screening-related LC mortality benefit observed in the trials had the largest effect on the ICER. ICERs ranged from $37,850–$277,950/QALY gained in the NLST-like simulation and $21,500–$104,350/QALY gained in the NELSON-like simulation when the LC mortality benefit approached the upper and lower 95% CI values, and when the benefit was assumed to decrease or continue after the trial (Fig. 2; incremental costs/LY Supplementary Fig. s4).

**Fig. 3:**
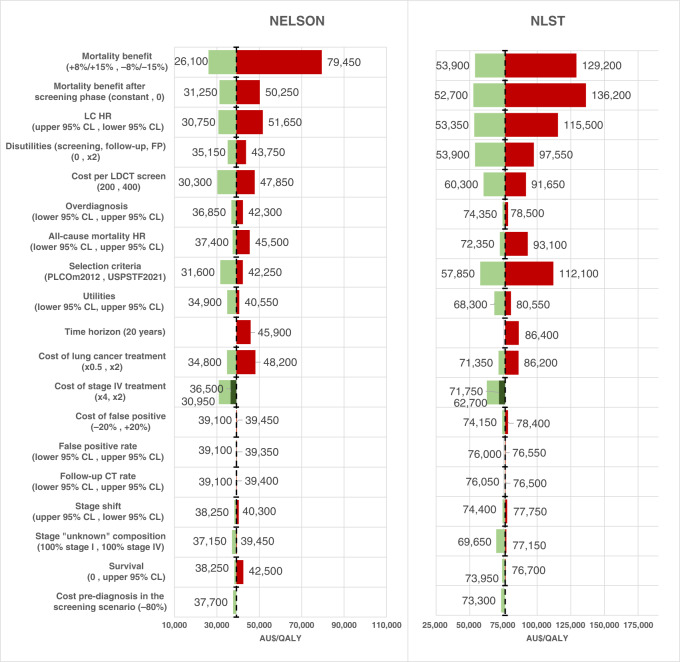
Estimated incremental cost-effectiveness ratios (ICERs) in one-way sensitivity analyses in relation to the base case estimated (dotted vertical lines) for the NELSON (left) and NLST (right) settings. CL confidence limit, HR hazard ratio, LC lung cancer, LDCT low-dose computed tomography, NELSON Nederlands–Leuvens Longkanker Screenings Onderzoek, NLST National Lung Screening Trial, USPSTF United States Preventive Services Task Force.

Variation in all other base-case parameters resulted in ICERs ranging from $53,350 to $115,500/QALY gained given the NLST trial and $30,300 to $51,650/QALY given the NELSON trial (see Table 2 and Fig. 3). Apart from the mortality benefit, among the parameters assessed, the LC mortality hazard ratio applied to eligible participants, disutilities related to screening, indeterminate and false-positive results, the cost per LDCT scan and the selection criteria had the greatest impact on the ICER (Fig. 3). The PLCO_m2012_ selection criteria was more cost-effective than both the base case and the USPSTF-2021 criteria (Table 2 and Fig. 3).

**Table 2:** One-way sensitivity analyses of variations in base-case assumptions. Incremental benefits and costs are presented per person participating in the programme.

Parameter	Value	Incremental cost $AU NLST/NELSON		Incremental benefit LYs NLST/NELSON		Incremental benefit QALY NLST/NELSON		ICER AU$/LYs NLST/NELSON		ICER AU$/QALY NLST/NELSON	
Base case	Base case	1434	1606	0.0375	0.0668	0.0188	0.0409	38,250	24,050	76,300	39,250
Mortality benefit	+8%/+15%	1434	1607	0.0495	0.0988	0.0266	0.0616	28,950	16,250	53,900	26,100
	−8%/−15%	1434	1605	0.0255	0.0350	0.0111	0.0202	56,250	45,850	129,200	79,450
Mortality benefit after the screening phase	0	1362	1483	0.0248	0.0494	0.0100	0.0295	54,900	30,000	136,200	50,250
	Constant	1492	1676	0.0502	0.0843	0.0283	0.0536	29,700	19,900	52,700	31,250
LC HR	Upper 95% CL	1499	1730	0.0504	0.0893	0.0281	0.0563	29,750	19,350	53,350	30,750
	Lower 95% CL	1386	1513	0.0279	0.0500	0.0120	0.0293	49,700	30,250	115,500	51,650
All-cause mortality HR	Upper 95% CL	1415	1569	0.0321	0.0572	0.0152	0.0345	44,100	27,450	93,100	45,500
	Lower 95% CL	1433	1609	0.0388	0.0701	0.0198	0.0430	36,950	22,950	72,350	37,400
Cost per LDCT screen	400	1723	1957	0.0375	0.0668	0.0188	0.0409	45,950	29,300	91,650	47,850
	200	1134	1240	0.0375	0.0668	0.0188	0.0409	30,250	18,550	60,300	30,300
Cost of LC treatment	x2	1621	1971	0.0375	0.0668	0.0188	0.0409	43,250	29,500	86,200	48,200
	x0.5	1341	1423	0.0375	0.0668	0.0188	0.0409	35,750	21,300	71,350	34,800
Cost of stage IV treatment	x4	1179	1266	0.0375	0.0668	0.0188	0.0409	31,450	18,950	62,700	30,950
	x2	1349	1493	0.0375	0.0668	0.0188	0.0409	35,950	22,350	71,750	36,500
Cost of false positive	+20%	1474	1613	0.0375	0.0668	0.0188	0.0409	39,300	24,150	78,400	39,450
	−20%	1394	1599	0.0375	0.0668	0.0188	0.0409	37,150	23,950	74,150	39,100
Cost pre-diagnosis in the screening scenario	−80%	1378	1542	0.0375	0.0668	0.0188	0.0409	36,750	23,100	73,300	37,700
False positive rate	Upper 95% CL	1439	1609	0.0375	0.0668	0.0188	0.0409	38,350	24,100	76,550	39,350
	Lower 95% CL	1429	1600	0.0375	0.0668	0.0188	0.0409	38,100	23,950	76,000	39,100
Follow-up CT rate	Upper 95% CL	1438	1611	0.0375	0.0668	0.0188	0.0409	38,350	24,100	76,500	39,400
	Lower 95% CL	1430	1599	0.0375	0.0668	0.0188	0.0409	38,150	23,950	76,050	39,100
Overdiagnosis	Upper 95% CL	1452	1680	0.0375	0.0668	0.0185	0.0397	38,700	25,150	78,500	42,300
	Lower 95% CL	1420	1543	0.0375	0.0668	0.0191	0.0419	37,850	23,100	74,350	36,850
Time horizon	20 years	1434	1606	0.0342	0.0580	0.0166	0.0350	41,950	27,700	86,400	45,900
	10 years	1434	1606	0.0232	0.0334	0.0090	0.0184	61,800	48,100	159,350	87,300
Stage shift	Upper 95% CL	1436	1611	0.0375	0.0668	0.0193	0.0421	38,300	24,100	74,400	38,250
	Lower 95% CL	1431	1600	0.0375	0.0668	0.0184	0.0397	38,150	23,950	77,750	40,300
Stage “unknown” composition	100% Stage I	1442	1617	0.0375	0.0668	0.0207	0.0435	38,450	24,200	69,650	37,150
	100% Stage IV	1420	1585	0.0375	0.0668	0.0184	0.0402	37,850	23,750	77,150	39,450
Survival (incidence scaling parameter)*	Upper 95% CL	1568	1878	0.0375	0.0668	0.0212	0.0442	41,800	28,100	73,950	42,500
	0	1396	1530	0.0375	0.0668	0.0182	0.0400	37,250	22,900	76,700	38,250
Selection criteria	PLCO_m2012_	1562	1909	0.0483	0.0951	0.0270	0.0604	32,350	20,050	57,850	31,600
	USPSTF-2021	1401	1606	0.0296	0.0634	0.0125	0.0380	47,350	25,350	112,100	42,250
Utilities	Lower 95% CL	1434	1606	0.0375	0.0668	0.0178	0.0396	38,250	24,050	80,550	40,550
	Upper 95% CL	1434	1606	0.0375	0.0668	0.0198	0.0421	38,250	24,050	72,400	38,150
	Recovery of Stage I	1434	1606	0.0375	0.0668	0.0210	0.0460	38,250	24,050	68,300	34,900
Disutilities (screening, follow-up, false positives)	0	1434	1606	0.0375	0.0668	0.0266	0.0457	38,250	24,050	53,900	35,150
	x2	1434	1606	0.0375	0.0668	0.0147	0.0367	38,250	24,050	97,550	43,750
Smoking cessation rate (screening/no-screening)	1.2	1434	1603	0.0399	0.0701	0.0202	0.0429	35,950	22,850	71,000	37,350
	1.5	1434	1603	0.0432	0.0750	0.0221	0.0457	33,200	21,350	64,900	35,100
	2	1434	1603	0.0485	0.0827	0.0252	0.0503	29,550	19,400	56,900	31,850

*CL* confidence limit, *HR* hazard ratio, *LC* lung cancer, *LDCT* low-dose computed tomography, *LY* life-years, *QALY* quality-adjusted life-year, *NELSON* NEderlands–Leuvens Longkanker Screenings ONderzoek, *NLST* National Lung Screening Trial, *USPSTF* United States Preventative Services Task Force.

*Population-based 5-year lung cancer survival was used to estimate the number of lung cancer cases.

The incremental costs in both trial settings were largely dominated by the cost per screen (accounting for ~74% of the incremental cost). Varying the cost of a LDCT scan from $200 to $400, resulted in a ~±20% change in the ICER (NELSON: $30,300–$47,850; NLST: $60,300–$91,650/QALY gained). Assuming a willingness-to-pay threshold of $30,000-$50,000/QALY gained, screening was cost-effective in the base-case scenario when the cost of a LDCT scan was $7–$135 given the NLST trial and $196–$424 given the NELSON trial.

The total cost of LC/person was relatively similar in the screening and no-screening scenarios ($7060 vs. $6870 NLST base case; $7000 vs $6640 NELSON base case), and the impact of varying all treatment costs by a factor of 1/2 or 2 on the ICER was moderate (from 6 to 22%). Doubling the healthcare costs of Stage IV disease or reducing the pre-diagnosis cost in the screening scenario by 80% resulted in a slightly more favourable ICER (4–7% change). The effect of varying the average cost of a false positive by 20% was also small (less than 3%).

Varying the overdiagnosis factor between the upper and lower limits of the 95% confidence intervals had a minor effect on the ICER (up to 8% change).

The population estimate of 5-year LC survival in the model was used to estimate the number of LC cases. Varying this parameter (and therefore varying the number of LC cases) in the one-way sensitivity analysis resulted in minor changes in the ICER. The effect of increasing LC survival to reproduce the upper 95% confidence limit of the LC incidence reported in the AIHW, was slightly more significant in the NELSON trial, increasing the ICER estimate to $42,500/QALY gained.

Reducing the time horizon to 20 years increased the ICER results by ~15%. Further reducing the time horizon to 10 years increased the ICER by more than 100%, to $159,350/QALY gained given the NLST or $87,300 given the NELSON scenario.

Given hypothetical increases in smoking cessation rates in the screening scenario (by a factor of 1.2–2), the ICER decreased by 7–26% ($71,000–$56,900/QALY gained) based on NLST and 5–19% ($37,350–$31,850 /QALY gained) based on NELSON.

## Discussion

We estimated the cost-effectiveness of LDCT screening for lung cancer to be $39,250 per QALY gained over a lifetime horizon by applying Australian costs and population-based lung cancer mortality and survival rates to the screening outcomes observed in the NELSON trial, using conservative assumptions for most parameters. The results obtained for the NLST setting were less favourable ($76,300/QALY gained) primarily because of the lower mortality benefit, which was the main driver of our model findings, and secondarily because of the higher rates of false positives. ICERs were extremely sensitive to the assumptions made regarding the lung cancer mortality benefit associated with screening during and after the active screening phase as observed in trials. Variation in this parameter resulted in the widest range of ICER values in one-way sensitivity analyses suggesting that cost-effectiveness of lung cancer screening in Australia is particularly contingent on achieving a mortality benefit that is at least equal to that observed in the trials. At an indicative ‘willingness-to-pay’ threshold in Australia of ~$30,000–$50,000, 15–60% of simulations in a probabilistic sensitivity analysis resulted in ICERs that could be considered cost-effective using NELSON-like screening parameters and outcomes.

This updated Australian evaluation for lung cancer screening is more favourable than previous Australian studies, including our 2018 evaluation which was based solely on the NLST setting [14]. Our NLST-like estimates of cost-effectiveness are more favourable in the current study due to a combination of the different time horizons used (lifetime vs 10 years), the population-derived LC mortality and survival rates used in this evaluation (as opposed to the trial rates used previously), and updated assumptions for other parameters. This updated evaluation is the first to incorporate Australian trends in smoking rates, which we simulated in a purpose-built, smoking prevalence forecasting model used to estimate both the number of Australians who currently smoke or have quit, and the competing risk of smoking-related all-cause mortality. We also incorporated updated, comprehensive health-system costs associated with lung cancer, estimated in a large population-based cohort study linked to routinely collected, administrative health databases [25].

A conservative approach was adopted in this analysis, reflected in several base-case assumptions. The first was that data on the cost of treating lung cancer did not capture immuno- and targeted therapies in use after 2016, which have substantially increased the cost of treating advanced and inoperable disease. Preliminary estimates suggest that the total healthcare costs for treating Stage IV lung cancer is almost twofold higher overall in 2021 compared to 2016 (noting that systemic therapy costs, which increased many-fold over this period, only account for a proportion of overall costs) [40]. Higher costs and lower survival associated with later stage disease are averted by screening, thereby potentially improving cost-effectiveness. In our analysis, doubling and quadrupling the total healthcare costs of Stage IV disease resulted in more favourable ICERs. However, even when quadrupling the total healthcare costs of Stage IV disease, variations in the cost of a LDCT scan had a large impact on the ICER. The cost of a LDCT scan could potentially be reduced in a large-scale screening programme, and our threshold analysis demonstrated that in the NELSON-like setting, lung cancer screening would be considered cost-effective in our base case if the scan price was set at $196, given a willingness-to-pay threshold of $30,000/QALY gained.

We conservatively assigned disutilities to the short-term psychological impact of screening and screening results, even though the evidence supporting a measurable effect on the utility scale following a positive or negative screening result is scarce. In the NLST and NELSON trial, there were no clinically relevant changes in quality-of-life detected in the SF-36 and SF-12 mental and physical component scores [29–31]. However, in the NELSON trial, the Impact of Event Scale (measuring lung cancer-specific distress) detected significant differences 2 months after a screening result [30, 31]. ICERs generated for the NLST-like setting were particularly sensitive to the degree of these disutilities, mainly due to the higher number of scans requiring follow-up CTs and further diagnostic work-up compared to the NELSON trial. Assigning zero disutilities in relation to screening and screening results, reduced the ICER in the NLST-like setting to from $76,300/QALY gained to $53,900. This implies that effective risk communication and access to appropriate support in relation to receipt of screening results is critical to reducing distress and improving cost-effectiveness. Additional conservative assumptions made in our base case included “no recovery” in quality of life for those diagnosed with lung cancer (i.e., the utility weights assigned at diagnosis were applied for the remainder of the survival period and corrected for age, resulting in a lower number of QALYs gained); and that eligible individuals who had quit smoking had the same all-cause mortality hazard ratio of those who were currently smoking (resulting in an increased number of deaths from other causes and in fewer QALYs gained).

Selecting a high-risk population is critical for optimising the balance of benefits and costs of a screening programme. In an exploratory analysis, we compared the results from two alternate selection criteria: the PLCO_m2012_ risk calculator and the USPSTF-2021 guidelines [6, 35]. PLCO_m2012_ is a lung cancer risk prediction model shown to yield a smaller number needed to screen to avert one lung cancer death compared to other criteria (i.e., NLST, NELSON, USPSTF) [41], and has been considered as an approach to define eligibility for a national targeted screening programme [12]. We estimated that selection of participants using a PLCO_m2012_ risk threshold of ≥1.51% was more cost-effective than the USPSTF-2021 criteria in both trial settings.

An exploratory analysis of smoking cessation, whereby higher smoking cessation rates were assumed in the screening scenario than the no-screening scenario, showed reductions in the ICER of 5–26%. However, we underestimated the costs of smoking cessation by assuming that screening itself had a positive effect on smoking cessation rates, without the additional cost of a cessation intervention. To maximise the benefits, targeted smoking cessation interventions may be, or are likely to be, necessary.

The screening input parameters of our model were closely related to the trial settings and so we did not have the flexibility to explore beyond these parameters. For example, recent lung cancer screening studies have reported more favourable stage shift distributions than the NLST and NELSON [42, 43], which will likely translate into better survival as well as averting the costs of treating inoperable disease. Furthermore, the mortality benefit that would accrue over long-term annual or biennial screens is potentially greater than what was observed in the trials and could result in a more favourable cost-effectiveness outcome for a full programme [44]. We could not simulate long-term annual or biennial screens directly because we modelled the mortality benefit as an independent parameter.

A potential limitation of our study was the assumption that LDCT screening had no effect on mortality from causes other than lung cancer. Specifically, we did not model actionable incidental findings [45–47]. Including incidental findings and the associated non-lung cancer mortality benefits may have had a favourable effect on mortality outcomes, however, the potential for overtreatment and complications in relation to these conditions would also need consideration. Similarly, we did not model adverse events, and in particular, fatal complications of diagnostic follow-up (although these are considered rare [3, 4]).

We also did not model variation in participation or screening adherence rates, both of which are key drivers of effectiveness and cost-effectiveness in national screening programmes. Further, the demographic profile of trial participants may not be representative of the eligible Australian population. The differential distribution of participants in the Australian population by characteristics known to be related to both lung cancer risk and screening participation, such as socioeconomic status, were not accounted for and may impact on screening implementation and effectiveness [48]. We also did not include any recruitment or programme overhead costs that would be needed for an equitable, population-based programme targeted at those who would obtain the greatest benefit, especially priority populations that may experience cultural, societal, psychological and/or physical barriers to participation [48].

Our results suggest that lung cancer screening with LDCT could be cost-effective in the Australian setting, dependent on achieving the mortality benefit observed in international trials. These findings contributed to a recent evaluation by the Australian Medical Services Advisory Committee, which in October 2022 recommended the establishment of a national screening programme in Australia. The effective implementation of a potential programme, and how well it is accepted and adopted by local health systems and high-risk communities, will be critical to its effectiveness and cost-effectiveness. If a national, risk-targeted lung cancer screening programme is rolled out in Australia, future economic evaluations using microsimulation models of the natural history of lung cancer to model beyond the direct evidence can be used to guide effective and cost-effective drivers of implementation success. Furthermore, updated evaluations can incorporate more contemporary data on improvements in lung cancer treatment, survival and quality of life as it becomes available, as well as potential variations in screening benefits by lung cancer histological sub-types.

## Supplementary information

Supplementary Material

## Data Availability

This research was completed using data collected through the 45 and Up Study (www.saxinstitute.org.au). The 45 and Up Study is managed by the Sax Institute in collaboration with major partner Cancer Council NSW; and partners: the Heart Foundation; NSW Ministry of Health; NSW Department of Communities and Justice; and Australian Red Cross Lifeblood. Data supporting the findings from this study are available from the Sax Institute, the NSW Department of Health, Cancer Institute NSW, and the Australian Bureau of Statistics, with data linkage conducted by the NSW Centre for Health Record Linkage (CHeReL; https://www.cherel.org.au). Restrictions apply to the availability of these data, which were used under license for the current study, and so are not publicly available. However, the 45 and Up Study is an open resource, accessible to any researcher upon application (https://www.saxinstitute.org.au/our-work/45-up-study/for-researchers/). All other data were obtained from publicly available sources online and previously published materials.

## Code availability

The computer code used for the model is proprietary. The authors can be contacted for further details.
