# Supplementary material for: Updated cost-effectiveness analysis of lung cancer screening for Australia, capturing differences in the health economic impact of NELSON and NLST outcomes
Source: Br J Cancer. 2022 Nov 3;128(1):91–101. doi: 10.1038/s41416-022-02026-8 (PMC9814515; doi:10.1038/s41416-022-02026-8)
Supplement: Supplementary file 1 — Supplementary Material [file 41416_2022_2026_MOESM1_ESM.docx]

Supplementary Material to:

**Updated cost-effectiveness analysis of lung cancer screening for Australia, capturing differences in the health economic impact of NELSON and NLST outcomes**

Silvia Behar Harpaz^1^*†, Marianne Weber^1^*, Stephen Wade^1^, Preston Ngo^1^, Pavla Vaneckova^1^, Peter Sarich^1^, Sonya Cressman^2^, Martin Tammemagi^3^, Kwun Fong^4,5^, Henry Marshall^4,5^, Annette McWilliams^6^, John Zalcberg^7^, Michael Caruana^1^*, Karen Canfell^1^*

**Contents**

[**General Model Overview** 2](#_Toc108009367)

[**Detailed assumptions regarding conditional probability of LC death.** 6](#_Toc108009368)

[**Proportion of population eligible for screening** 6](#_Toc108009369)

[**LC mortality hazard ratios** 7](#_Toc108009370)

[**Detailed assumptions regarding the screening-related LC mortality benefit after the screening phase of the trials.** 8](#_Toc108009371)

[**Detailed assumptions regarding costs** 10](#_Toc108009372)

[**Detailed assumptions regarding stage shift and stage “unknown.”** 12](#_Toc108009373)

[**Utility Weights and calculation of QALYs** 13](#_Toc108009374)

[**One-Way Sensitivity Analyses and Probabilistic Sensitivity Analysis** 16](#_Toc108009375)

[**References** 22](#_Toc108009376)

# **General Model Overview**

The cost-effectiveness of lung cancer screening with LDCT in Australia was assessed by applying Australian costs, mortality, mortality hazard ratios by smoking status, and survival data to the outcomes observed in the NELSON and NLST trials, which have shown a mortality benefit of ~20% and a cancer stage distribution shift when screening individuals with a history of heavy tobacco exposure.

A simplified, multi-cohort, deterministic model was implemented in R^[1,2]^, where the all-cause and lung cancer (LC) mortality rates by sex, age and smoking status, and a LC mortality benefit during screening, were used to estimate the number of LC deaths and the life years gained in both screening and no-screening scenarios.

Australian costs of treatment and LDCT scans were applied to estimate the incremental cost, incremental effectiveness and ICER for participants of a screening trial set in the Australian population.

Note the approach adopted in our study, goes “backwards” from mortality to total incidence and incidence by stage. This approach has the advantage of using the main output from the trials – the mortality benefit – as a direct input which allows a straightforward and simple calculation of the life years gained. This “backwards” thinking reduces the uncertainty in the denominator of the CER since the large uncertainties around the survival by stage, stage shift, “unknown” stage composition, and other parameters do not play any role in the LYs (when calculating QALYs they have a limited influence because of small differences in disutilities corresponding to different stages at diagnosis). These parameters are used mainly to estimate the incremental cost of treatment. However, since the total incremental cost is mostly dominated by the cost of screening, uncertainties around these parameters, result in a relatively small uncertainty about the CER.

The simulation starts at a given calendar year (2020) with an initial number of participants (N_0,sex,age,status_), for each age, sex and smoking status (smoker or ex-smoker), based on the trial eligibility criteria applied to the 45 and Up Study cohort.

In the no-screening scenario, the all-cause mortality rate (by age, sex and smoking status) was applied at each time step to find the number of participants alive at the following time step. The all-cause mortality rate was assumed to be equal to the LC mortality rate + other-cause death rate.

In the screening scenario, the timeline was divided into three sections (Figure s1):

1. The screening phase ($T_{0}\leq t\leq T_{1}$): All eligible participants alive were scanned with LDCT at the timepoints defined in the trial. The LC mortality benefit (*b*) observed in the trial was applied, resulting in a modified LC mortality rate.

2. After trial phase $(T_{1}\leq t\leq T_{2})$: The LC mortality benefit decreased and reached 0 at time $T_{2}$. More details about the assumptions regarding the mortality benefit after the trial ends, can be found below.

3. $T_{2}\leq t\leq T_{3}$: The LC mortality rate was assumed to be equal in both scenarios. Life years in both scenarios were counted up to the time horizon ($T_{3}$).

The cost of treatment varies significantly according to the cancer stage at diagnosis. Therefore, to estimate the costs in both scenarios it was necessary to calculate the number of cases diagnosed in each stage (Figure s2). This was determined by first estimating the number of LC cases diagnosed by age, sex, and smoking status, and then applying an appropriate stage distribution for the scenario (screening or no-screening).

The number of LC cases diagnosed (excluding over-diagnosed cases) was assumed to be equal in the screening and no-screening scenarios. The number of cases at each time point, by sex, age, and smoking status, was obtained by dividing the (sex, age and smoking status-specific) number of LC deaths amongst participants by the conditional probability of death given a LC diagnosis – which was approximated by one minus the 5-year relative survival (by age and sex). For the screening scenario, the number of diagnosed cases during the trial was multiplied by a constant factor to account for overdiagnosis, using proportions estimated from the trials. Specifically, the overdiagnosis rates reported in both trials use the number of screen-detected cases as denominators, i.e., they report the fraction of over-diagnosed cases out of the number of screen-detected cases. In our study, we did not differentiate between screen-detected and not screen-detected cases in the screening arm. Therefore, the overdiagnosis rate used the total number of cases in the screening arm as denominator. The NELSON trial reported an overdiagnosis rate of 8.9%. The number of screen detected cases in the screening arm was 203, while the total number of cases in the screening arm was 344. Therefore, the overdiagnosis rate using the total number of cases as denominator for the NELSON trial is: 0.089*203/344 = 0.0525 (approximately 5.3%). The extended follow up of the NLST trial reported an overdiagnosis factor of 3%. The total number of LC cases in the LDCT arm was 1060, from which 649 were screen-detected. Thus, the corrected overdiagnosis factor is 0.03*649/1060 = 0.0184 (which was rounded up to 1.9%). The overdiagnosis rate was applied during the same period in which the mortality benefit was applied (the screening phase + 3 or 4 years).

In addition, a false positive rate (based on trial results) was applied at each screen time point.

The stage distribution at diagnosis by sex and age (from Australian, AIHW data) was applied to find the number of cases diagnosed at each cancer stage (I, II, III, IV or unknown). For the screening scenario, the stage shift observed in the different screening trials was applied to the stage distribution at diagnosis (during the trial), as well as a correction for ‘unknown’ stage which would not be a likely classification amongst screen-detected lung cancers. Further details regarding the shift are provided below.

Screening with LDCT allows earlier detection of LC cases, resulting in a stage shift towards earlier cancer stages and therefore better survival rates. However, LDCT screening does not prevent lung cancer and (excluding over-diagnosed cases) does not change the total number of cases diagnosed. In this analysis, the incidence is identical across screening and no-screening settings, however screening with LDCT results in earlier detection and therefore higher incidence at younger ages; giving rise to the notion of ‘lead time’ which is the difference between when a screen-detected cancer is diagnosed compared to a counterfactual usual-care setting without screening. Lead time is an important consideration in analysis of survival, and hence our mortality benefit is directly informed by well-designed and powered randomized control trials with cause-specific mortality as the end point. The timing of costs would differ across screening and no-screening scenarios in a real trial – an effect we do not account for – however any impact of the differences on the resulting ICERs would only be noticeable at unrealistically high discount rates. For simplicity, the lead time (the time between the early detection of cancer by screening and its usual clinical presentation) was not directly considered in this model, and the incidence at each time point was assumed to be the same in both scenarios. Nevertheless, the lead time and early detection in the screening scenario were indirectly taken into account through the assumed LC mortality benefit and the stage shift, which result in the same total number of incident cases, a reduced number of LC deaths, and the same total costs that would have been obtained if a lead time was applied.

The average health system costs of LC by stage were estimated from a previous analysis of the 45 and up study ^[3]^. The average cost associated with LDCT screening and the average cost of false positive cases was based on the Queensland Lung Cancer Screening Study ^[4]^. Indeterminate screening tests which required a repeat CT scan were accounted in the model as a follow-up CT rate, which only increased the average cost of a LDCT scan. The false positive rate (and false positive cost) refered to positive CT scans which required further non-CT workup. Follow-up CT and false positive rates were based on trial results.

A constant discount rate of 5% was applied to the costs and benefits.

Further details regarding the assumptions are given in the following sections.

**Figure s1.** Model Overview Part 1: calculation of life years (LYs)

**Figure s2.** Model overview part 2: calculation of costs


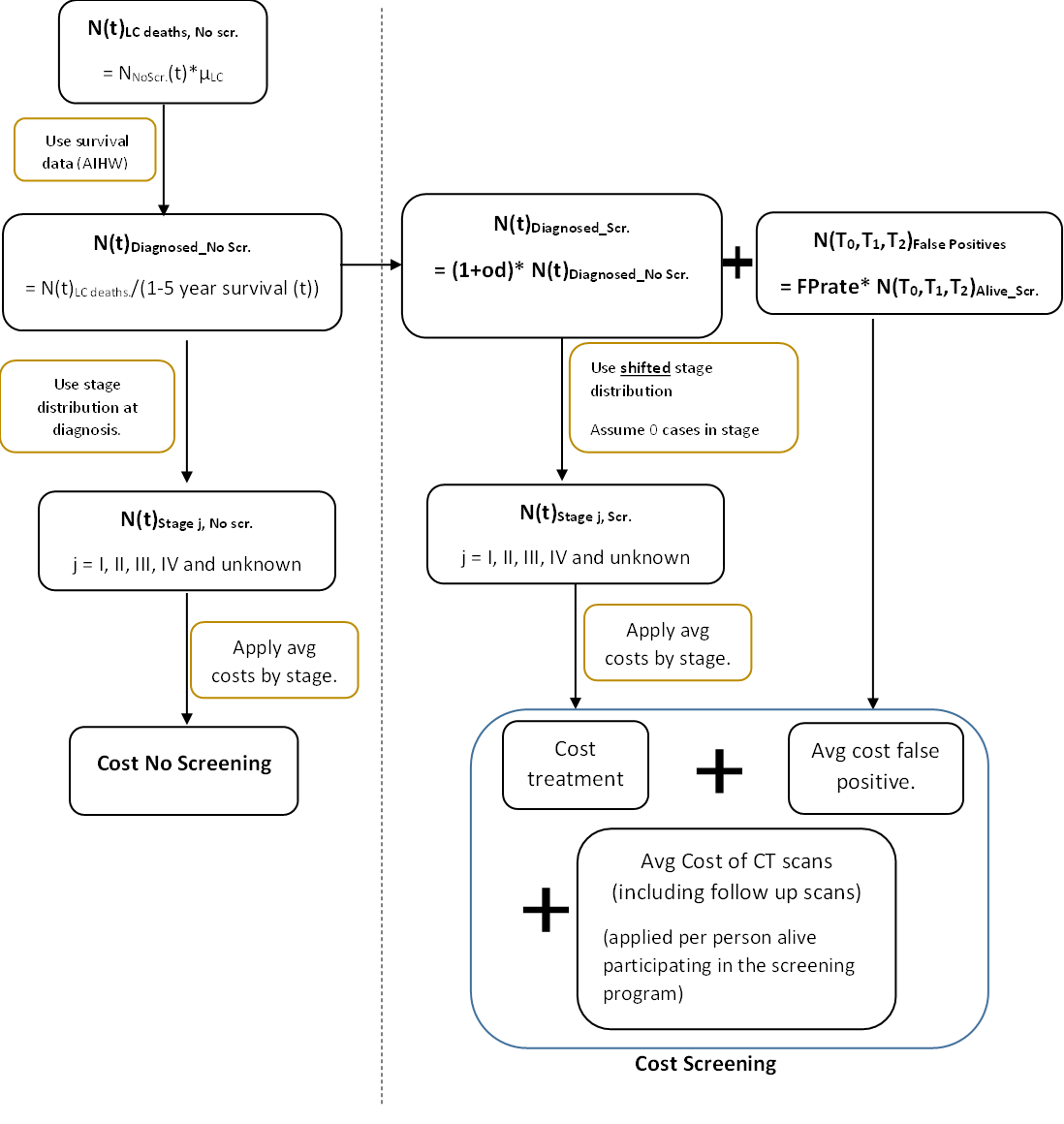


R packages included in the code: : tidyverse ^[5]^ and plyr ^[6]^ for the main code; ggplot2 ^[7]^, gridExtra ^[8]^, sn ^[9]^, and EnvStats ^[10]^ to generate the plots and the PSA.

# **Detailed assumptions regarding conditional probability of LC death.**

The number of lung cancer incident cases (by sex and age) was given by:

1. $N_{Incident}= \frac{N_{LCdeaths}}{P\left( {death}/{diagnosis} \right)}$,

where $P\left( {death}/{diagnosis} \right)$ was the conditional probability of LC death given a LC diagnosis and was approximated in this model by $P\left( {death}/{diagnosis} \right)\cong$ 1 - 5-year relative survival probability (taken from the AIHW Australian Cancer Database 2016^[11]^).

Figure s3 shows a comparison of the incidence according to the AIHW Australian Cancer Database 2016 to the results obtained by applying equation 1. The incidence was underestimated in our model. The effect of varying the survival inputs between the limits which reproduce the 95% confidence interval of the incidence was estimated in a sensitivity analysis.

**Figure s3** Age specific lung cancer incidence rate form men (left) and women (right), according to the AIHW Australian Cancer Database 2016 compared to the model values.


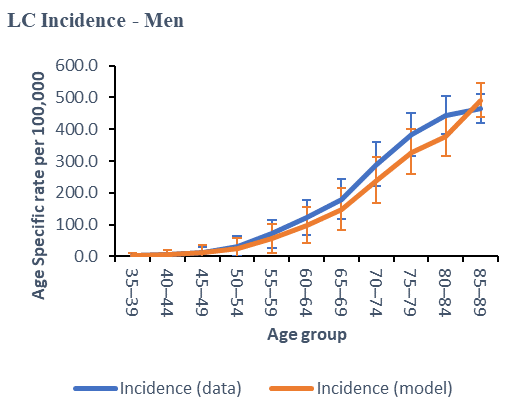

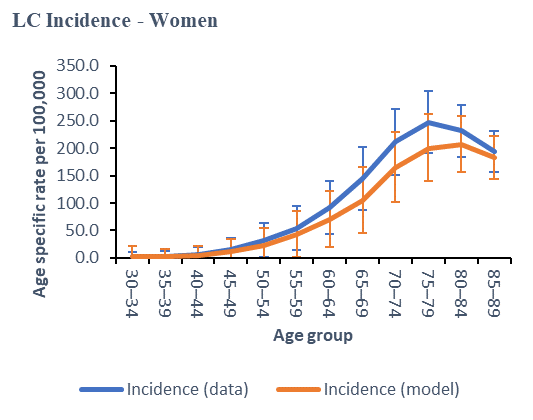


# **Proportion of population eligible for screening**

The trials analysed in this work involved participants with a history of heavy tobacco exposure. The NLST trial enrolled participants between 55 and 74 years of age, had a history of cigarette smoking of at least 30 pack-years, and, if former smokers, had quit within the previous 15 years. NELSON’s selection criteria involved current or former smokers (who had quit not more than 10 years ago), who had smoked more than 15 cigarettes a day for more than 25 years or more than 10 cigarettes a day for more than 30 years. Since smoking behaviours are not systematically collected in any population-wide database in Australia, there is currently no way of accurately ascertaining the proportion of Australian’s who would be eligible for lung cancer screening at any one time. To estimate the proportion eligible for the purposes of modelling, we applied the screening selection criteria to a large, population-based Australian cohort study, the Sax Institute’s *45 and Up Study*^[12]^. Our previous publication, in which we validated the PLCOm2012 risk tool and the NLST criteria in the 45 and Up Study, provides detailed descriptive information on the characteristics of participants with a history of smoking who did or did not meet eligibility criteria^[13]^. We leveraged this previous work to ascertain the proportion of participants eligible for screening for use in the current model.

Briefly, the cohort included 267,153 residents of New South Wales aged ≥45 years at baseline (2006-2009), randomly sampled from the Services Australia (formerly the Department of Human Services) enrolment database and followed up via linkage to routinely collected administrative health databases (conducted by the Centre for Health Record Linkage: <https://www.cherel.org.au/> and accessed from the Secure Unified Research Environment). Study participants’ identifiers were linked to population-wide health databases: 1) the NSW Cancer Registry (Jan 1994 – Dec 2013), which receives all notifications of cancer (except non-melanoma skin cancer) for NSW residents; 2) the Register of Births, Deaths, and Marriages (Jan 2006 – Dec 2015), which contains all death notifications; and 3) The Cause of Death Unit Record File (Jan 2006 – Dec 2015). Records in these datasets were probabilistically linked^[14]^ by the Centre for Health Record Linkage using a best practice approach in privacy preserving record linkage^[15]^ and the open source probabilistic record linkage software Choice Maker. The probabilistic matching process is known to be highly accurate (false-positive and false-negative rates <0.4%) and has been described in more detail elsewhere^[16].^ Participants who migrated out of NSW were lost to follow-up as the linked health records were for NSW only, but the proportion was likely to be small. People 80+ years of age and residents of remote areas were oversampled, and the overall response rate was ~18% of invitees.

The variables used to ascertain participants’ eligibility for screening according to each selection criteria at baseline in the 45 and Up Study included smoking status, number of cigarettes smoked per day, number of years smoked, and age. Participants with missing information on smoking behaviours, record linkage errors, or inconsistent death information were excluded, as were participants with a registry-notified cancer prior to baseline, with 222,472 participants remaining for analysis, including 16,059 participants classified as current smokers at baseline, 75,358 former smokers, and 131055 never smokers.

As discussed in our previous paper ^[13]^ due to possible selection bias, the 45 and Up Study cohort is likely to have reduced representation of population groups who have more risky health behaviours and higher smoking rates, such as those who are mentally or physically ill, or marginalised for some other reason. Furthermore, the survival bias inherent in cohort studies of older age groups and especially samples of long-term smokers, mean that our estimates of the proportion eligible for screening are likely to be conservative. Conversely, individuals with the heaviest smoking history in the population are those most likely to have multiple, or serious comorbidities, putting them in a subgroup for which screening may be inappropriate. Specifically, only those with both reasonable life expectancies and suitability for lung cancer treatment will benefit from screening. Taking all these factors into account, the prevalence estimate from this cohort is likely to represent a somewhat conservative, but justifiable, approximation of the number of individuals who would be eligible for screening in the population as a whole.

# **LC mortality hazard ratios**

Table s1 shows the LC mortality hazard ratios (HRs) of participants by selection criteria, estimated from the Australian 45 and Up Study cohort using the dataset described above and using the statistical methods detailed in our previous publication: Weber et al., 2021^[17]^. All HRs were calculated with never-smokers as the reference group.

**Table s1** LC mortality hazard ratios (HR) of participants by smoking status for each trial selection criteria, estimated form the Australian 45 and Up Study cohort.

|  | **LC mortality HR (95% confidence interval)** | |
| --- | --- | --- |
| Smoking status (reference Never) | **Current** | **Former** |
| All participants | 19.75 (95% CI 16.09-24.25) | 5.73 (95% CI 4.79-6.84) |
| NELSON eligible participants | 26.86 (95% CI 20.13 - 36.86) | 18.47 (95% CI 13.79-24.73) |
| NLST eligible participants | 30.12 (95% CI 22.22-40.83) | 16.91 (95% CI 12.65-22.6) |

To calculate the specific age-specific LC mortality rates of eligible participants, smoking prevalence data generated by a model of life-course smoking behaviour for the Australian population was used to solve equation 2 and find the age-specific LC mortality rates of never smokers in Australia ($\mu_{never}$).

1. $\mu_{All}=f_{never}\mu_{never}+f_{smoker}{HR}_{smoker}\mu_{never}+f_{ex-smoker}{HR}_{ex-smoker}\mu_{never}$,

where $\mu_{All}$ were the age-specific LC mortality rates of the Australian population in 2016 as reported by the Australian Institute of Health and Welfare, and $f_{status}$ were the proportions of individuals by smoking status (never, former, current) in the population in the same year.

The age-specific mortality rates of eligible participants by smoking status (current and former) were found by multiplying $\mu_{never}$ by the corresponding hazard ratio.

A similar calculation was performed to find the age-specific all-cause mortality rates of eligible participants in the years 2016-2066 using ABS projected mortality rates^[18]^ and the projected proportions of individuals by smoking status in each year.

Table s2 shows the posterior all-cause mortality hazard ratio of individuals who smoke from the Australian smoking prevalence model, informed by national smoking survey data and the 45 and Up study^[12,19]^. For simplicity, it was assumed that all screening eligible 45 and Up Study participants had a similar hazard ratio of all-cause mortality (and therefore the same age-specific all-cause mortality rates) as individuals in Australian population who were currently smoking. The observed differences between the overall cohort and the eligible subgroups in the lung cancer-specific mortality hazard ratio (see Table s1) suggest that this assumption may lead to an underestimate of the all-cause mortality risk of screening eligible individuals who have a history of heavy tobacco use, and hence overestimate the life years gained by screening.

In our base case, we used the average LC hazard ratio for all the eligible individuals with a history of former smoking, independent of time since quitting, and the LC hazard ratio was assumed to be constant over time.

**Table s2.** Hazard Ratios (HR) and 95% confidence intervals (CI) for death from any cause among individuals that smoke compared to those who never smoked, from the Australian smoking prevalence model.

|  | Men | | | Women | | |
| --- | --- | --- | --- | --- | --- | --- |
| Age | **HR** | **95% CI** | | **HR** | **95% CI** | |
| 45-49 | 3.19 | 2.3 | 4.41 | 3.53 | 2.6 | 4.79 |
| 50-54 | 3.24 | 2.39 | 4.38 | 3.74 | 2.95 | 4.74 |
| 55-59 | 3.71 | 2.93 | 4.69 | 3.91 | 3.16 | 4.86 |
| 60-64 | 4 | 3.23 | 4.94 | 3.72 | 3.1 | 4.47 |
| 65-59 | 4.6 | 3.8 | 5.56 | 3.38 | 2.83 | 4.03 |
| 70-74 | 4.24 | 3.33 | 5.4 | 3.58 | 2.97 | 4.3 |
| 75-80 | 2.45 | 1.81 | 3.32 | 2.56 | 2.06 | 3.19 |
| 80-84 | 2.48 | 2 | 3.08 | 1.99 | 1.65 | 2.41 |
| 85-89 | 1.31 | 0.92 | 1.86 | 1.27 | 0.91 | 1.78 |
| 90-99 | 0.9 | 0.51 | 1.57 | 1.26 | 0.74 | 2.12 |

# **Detailed assumptions regarding the screening-related LC mortality benefit after the screening phase of the trials.**

The base case assumptions in this model were based on the NLST and NELSON trials settings and outcomes.

In 2011, the NLST study reported a 20% (95% CI: 6.8% -26.7%) reduction in lung cancer mortality in the LDCT arm after a median of 6.5 years follow up (about 4.5 years after the final scheduled screen)^[20].^ However, an extended follow-up of the NLST showed a dilution-adjusted LC mortality relative risk (RR) of 0.89 (95% CI: 0.80-0.997) after a median of 12.3 years follow-up (RR=0.92 with 95% CI: 0.85-1.00 ignoring dilution effect)^[22]^. Note that with the extended follow-up some of the patients in whom cancer did not develop until after the last screen, might not have benefited from the trial (dilution effect). However, the dilution-adjusted analysis included only those cancer deaths for which the corresponding time of cancer diagnosis was close enough to the end of the screening phase of the trial, meaning that the reduction in mortality benefit was mostly a result of LC deaths which were only delayed by the trial instead of being avoided. Since the LC cumulative incidence relative risk across arms in the NLST first became nonsignificant at year 6 (4 years after the last screen), for the dilution adjusted analysis only deaths with diagnosis up to study year 6 were included.

The NELSON screening trial^[21]^ reported a cumulative rate ratio for death from lung cancer at 10 years among men of 0.76 (95% CI: 0.61 – 0.94) in the screening group as compared to the control group. The 10 year follow-up in the NELSON trial consisted of a screening phase of 5.5 years + additional follow-up of 4.5 years.

Our model does not need to accurately describe the LC incidence and mortality as a function of time during and after the trial, but rather to give a reliable estimate of the total number of LC deaths avoided by screening and the incremental cost of the trial setting. For this purpose, the mortality benefit was assumed to be constant during the screening phase and to decline linearly to 0 between T_1_ and T_2_ (the period between the last screen and a cutoff time T_2_). The value of the constant and T_2_ were set to reproduce the average mortality benefit observed in the trials (CER results were also examined using alternative values and assumptions for the mortality benefit in a sensitivity analysis).

As noted above, lead time for screened cases was not directly considered in this model, and the incidence at each time point was assumed to be the same in both scenarios. However, lead time was indirectly accounted for through the assumed LC mortality benefit and stage shift, which result in the same total number of incident cases, reduced number of LC deaths, and the same total costs that would have been obtained if a lead time was applied.

Two sets of parameters were analysed in two different base cases (corresponding to the NLST and NELSON trials). For the NLST base case the constant mortality benefit during the screening phase was assumed to be 0.8, declining linearly to zero three years after the last screen. The resultant LC mortality RR, accounting for all LC deaths up to year 6, was 0.86, and up to year 12, 0.94. Considering that in our simplified model the LC deaths were counted at the year of diagnosis, these results are consistent with the RR of 0.89 and 0.92 observed in the extended follow up of the NLST trial.

In the NELSON base case, a conservative constant value of 24% was assumed for the mortality benefit during the trial, declining to zero linearly 4 years after the last scan, resulting in a cumulative LC mortality RR of 0.82 at year 10, consistent with the results observed in the trial.

The ICER values obtained by varying the assumptions in the mortality benefit to approximate the 95% confidence intervals of the cumulative mortality RR reported in the trials are shown in Figure 2 (for Incremental Cost/QALY; main analysis). The ICERs for Incremental Cost/ LY gained are shown in Figure s4.

**Figure s4** . Incremental cost (in AU$/person) vs incremental LYs/person for the NLST and NELSON settings, obtained by varying the assumptions related to the mortality benefit.


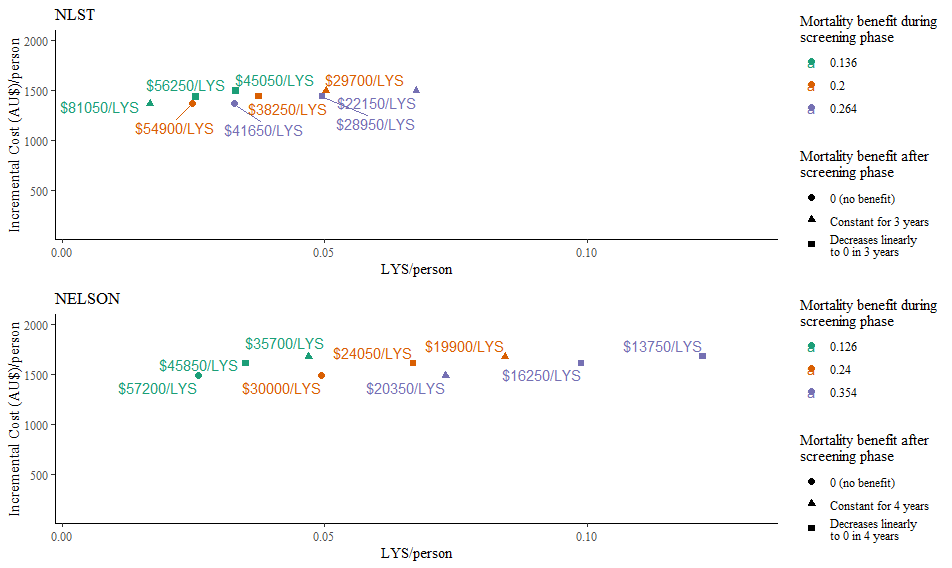


# **Detailed assumptions regarding costs**

The average health services cost of LC by stage was estimated from the 45 and up study by extent of disease at diagnosis (classified by the NSW cancer registry based on stage) and phase of care. An initial treatment cost was applied to all LC cases. For cases who survived, an additional cost for continuing care was applied, while for cases who died, a fraction of the continuing care cost (depending on the mean survival time by stage) and a terminal care cost were applied. Table s3 summarises the excess costs by phase of care and selected tumor characteristics, for participants in the 45 and Up Study diagnosed with LC between 2006-2013 as reported by Goldsbury et.al^[3]^.

The excess costs reported in Goldsbury et.al^[3]^ were estimated from records of individuals’ inpatient hospital episodes, ED presentations, and subsidised prescription medicines and medical services recorded in the PBS and MBS respectively. The “average case” excess cost for case-control groups were calculated from 1 year before to 3 years after diagnosis. More details regarding the average excess costs applied in this study, are given in [3].

Let $P_{j}$ be the cost prior to diagnosis for stage *j*, let $I_{j}$ be the cost of the initial treatment phase, let $C_{j}$ be the cost of the continuing care phase, and $T_{j}$ the cost of the terminal care phase. Then, the cost of stage *j* for cases who did not die (survived $n\geq N$ years) was given by:

1. ${Cost}_{j,n\geq N}=P_{j}+I_{j}+\left( N-1 \right)*C_{j}$.

For cases who died, if the case died in less than 1 year, then the cost of stage *j* was given by:

1. ${Cost}_{j,n<1}=P_{j}+T_{j}$, and if the case survived $n$ years ($1\leq n<N$), then the cost was given by:
2. ${Cost}_{j,1\leq n<N}=P_{j}+I_{j}+\left( n-1 \right)*C_{j}+T_{j}$.

Finally, the 1, 2, 3, 4 and 5 year survival data by stage was applied to calculate the proportion of cases who died and who survived for each time period, and these proportions were used to calculate a weighted average cost for each stage.

In our calculations, the costs of stages I and II were approximated by the cost of “Localised” stage, the cost of stage III by “Regional” stage, and the cost of stage IV by “Distant Metastases”.

The calculated “average case” costs to 3 years by phase, obtained by applying the 1, 2 and 3-year survival data, were $51,300, $61,039, $57,549, $54,324 and $33,985 for stages I, II, III, IV and “unknown” respectively, consistent with values reported by Goldsbury et al. ($51,531, $57,905, $54,543 and $36,462 for localised, regional, distant metastases and unknown stages respectively).

For our base case, the average costs were extended to 5 years, resulting in $62,327, $69,235, $63,436, $56,701 and $38,388 for stages I, II, III, IV and unknown respectively (all costs were given in 2021 Australian dollars).

Note that in the screening scenario, the excess costs in the year prior to diagnosis were expected to be lower than the values reported by Goldbsury et al., given that the values captured the cost of case-finding in an unscreened population. However, our base case assumed the same average costs in both scenarios.

Moreover, the average cost of treatment for stage IV is likely to have increased substantially since the study by Goldsbury et al., for which costs were captured up to the end of 2016, prior to the availability of targeted and immunotherapies for people with advanced LC at diagnosis.

The effect of varying all the costs was studied in the sensitivity analyses.

**Table s3** Summary excess costs by phase of care and selected tumor characteristics, for eligible incident lung cancer cases diagnosed 2006-2013, from Goldsbury et.al. ^[1]^

|  | **Excess costs in the year prior to diagnosis** | | **Initial treatment phase (if case survived >1 year)** | | **Continuing care phase (if case survived >2 years)** | | **Terminal care phase (if case died)** | |
| --- | --- | --- | --- | --- | --- | --- | --- | --- |
| **NSCLC by stage** | **Mean cost** | **Standard deviation** | **Mean cost** | **Standard deviation** | **Mean cost** | **Standard deviation** | **Mean cost** | **Standard deviation** |
| **Localised** | **$6,010** | $19,023 | **$27,679** | $21,590 | **$4,705** | $15,630 | **$53,679** | $46,751 |
| **Regional** | **$4,196** | $16,446 | **$30,989** | $25,507 | **$5,995** | $24,781 | **$42,718** | $43,229 |
| **Distant metastases** | **$2,427** | $15,216 | **$32,857** | $27,983 | **$18,729** | $25,769 | **$45,301** | $32,104 |
| **Unknown** | **$4,606** | $16,779 | **$10,859** | $17,243 | **$9,272** | $19,414 | **$25,324** | $32,273 |

# **Detailed assumptions regarding stage shift and stage “unknown.”**

The stage distribution in each trial is shown in Table s4. Both trials showed a clear shift towards stage I.

**Table s4** Proportions in each stage by arm in lung cancer screening trials. Based on Aberle D.R. et. al. ^[20]^ and de Koning HJ ^[21]^

| **Trial** | **Arm** | **Stage I** | **Stage II** | **Stage III** | **Stage IV** | **Stage Unknown** |
| --- | --- | --- | --- | --- | --- | --- |
| **NLST** | chest X-ray | 289 (30.7%) | 74(7.9%) | 231(24.5%) | 335(35.6%) | 12(1.3%) |
|  | LDCT | 520(49.1%) | 73(6.9%) | 221(20.8%) | 226(21.3%) | 20(1.9%) |
| **NELSON** | usual | 41(13.5%) | 30(9.9%) | 77(25.3%) | 139(45.7%) | 17(5.6%) |
|  | LDCT | 139(40.4%) | 29(8.4%) | 61(17.7%) | 92(26.7%) | 23(6.7%) |

To model the observed shift towards stage I, we assumed that fractions *f*_II_, *f*_III_ and *f*_IV_ transitioned from stages II, III and IV respectively to stage I. Stage unknown was assumed to remain unchanged. During the screening phase a constant stage shift was applied (using the stage shift factors from the trials). After the screening phase, for the next 3 (NLST) or 4 (NELSON) years the stage shift factors change linearly (as the mortality benefit), going back to the unmodified stage distribution at the end of this period.

Let *n*_I_, *n*_II_, *n*_III_ and *n*_IV_ be the number of cases in stages I, II, III and IV in the no-screening scenario. The corresponding number of cases in the screening scenario was given then by:

1. ${n^{'}}_{I}=n_{I}+n_{II}f_{II}+n_{III}f_{III}+n_{IV}f_{IV}$

$${n^{'}}_{II}=n_{II}\left( 1-f_{II} \right)$$

$${n^{'}}_{III}=n_{III}\left( 1-f_{III} \right)$$

$${n^{'}}_{IV}=n_{IV}\left( 1-f_{IV} \right)$$

The average values of $\left( 1-f_{i} \right)$ were found from Table s4 as the ratio of fraction of cases in stage *i* in the LDCT arm to the control arm.

Note that the more complicated set of equations that would be obtained by explicitly adding the possible intermediate transitions (from stage IV to stages III and II and from stage III to II) could be rewritten to take the simpler form given in (6).

However, the Australian stage distribution at diagnosis presents a considerably larger proportion of cases in stage “unknown” compared to the trials (see Figure s4 (a)). If screened cases undergo complete diagnostic work-up after being detected by LDCT, few “unknown” cases are expected in the screening phase. Therefore, before applying the stage shift described above, an assumption was made regarding the composition of the stage “unknown” in the Australian population stage distribution.

The proportions of cases in stage “unknown” likely to belong to stages I, II, III and IV ($p_{I}$, $p_{II}$, $p_{III}$ and $p_{IV}$) was estimated by comparing the survival data of stage “unknown” to the other stages. Let $S_{i}$ be the relative survival of stage $i$. Then:

1. $S_{unkonwn}=p_{I}S_{I}+p_{II}S_{II}+p_{III}S_{III}+p_{IV}S_{IV}$

In our base case, the best fit result of equation 7 to all the available 1, 2, 3, 4 and 5-year survival data (constrained to $p_{I}+p_{II}+p_{III}+p_{IV}=1$ and $p_{I},p_{II},p_{III},p_{IV}>0$) was used. The approximate composition of the stage “unknown” found as described above is ~60% stage IV, ~21% stage III, ~12% stage II and ~7% stage I. The extreme cases in which all the unknown cases are stage I or all of them are stage IV were studied in sensitivity analyses.

Figure s5 shows (a) the Australian stage distribution at diagnosis compared to the stage distributions observed in the usual arms of the trials, (b) the Australian stage distribution obtained after “redistributing” the unknown cases and after applying the stage shift, (c) the final Australian stage distribution after applying the stage shift compared to the stage distributions observed in the LDCT arms of the trials.

**Figure s5:** a) Australian stage distribution at diagnosis compared to the stage distributions observed in the usual arms of the trials, (b) Australian stage distribution obtained after “redistributing” the unknown cases and after applying the stage shift, (c) final Australian stage distribution after applying the stage shift compared to the stage distributions observed in the LDCT arms of the trials.


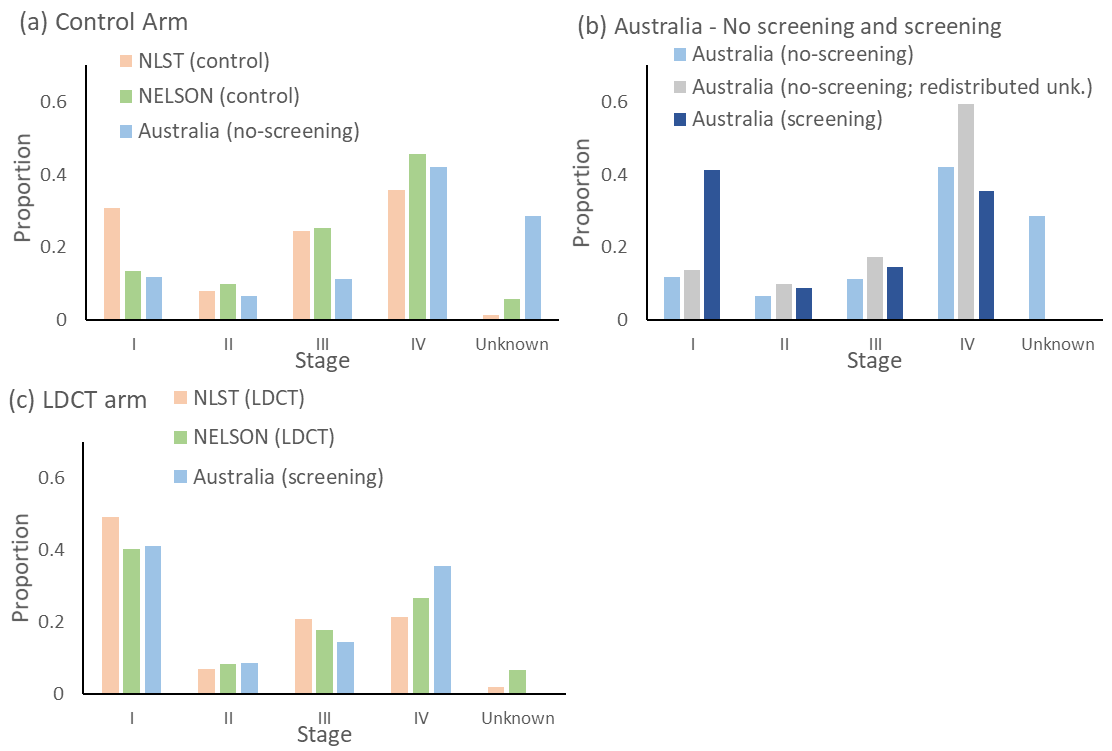


# **Utility Weights and calculation of QALYs**

Quality of life was approximated by a utility, which is a measure of a preference for a given health state, scaled from 0 to 1 (where 0 equals death and 1 equals perfect health).

The baseline utilities for participants who are eligible for the screening program were estimated from the 45 and Up data^[23]^. The baseline utilities for men and women eligible according to the NLST selection criteria were 0.787 (95% CI 0.769-0.800) and 0.749 (95% CI 0.740-0.757) respectively. The baseline utilities obtained for the NELSON selection criteria were 0.779 (95% CI 0.772-0.786) for men and 0.757 (95% CI 0.750-0.765) for women. A drop in utility of 0.01 was assigned at age 70-79, and 0.04 at age 80+ ^[24]^.

A small temporary disutility was applied for lung cancer screening itself (0.02 lasting for 2 weeks). The same disutility but for a longer period of 3 months was applied to indeterminate results which required a follow-up CT. False positive results were assumed to have the same lower utility as stage I LC for a period of 3 months.

Utility weights for those with lung cancer by stage at diagnosis were taken from the Cancer Care Outcomes Research and Surveillance (CanCORS) Study ^[25]^ and are presented in Table 1 (main text). We assumed that the utility weight assigned at diagnosis declined by 0.01 at age 70-79 years and 0.04 at age 80+.

The difference in QALYs between the screening and no-screening scenario was calculated as follows:

$${QALY}_{screening}=\sum_{t} \left( {N(t)}_{\begin{aligned} alive. \\ no LC, \\ screening \end{aligned}}\times U_{b}+\sum_{i} {N(t)}_{\begin{aligned} dead, \\ stage i, \\ screening \end{aligned}}\times{\tau_{i}\times U}_{i}+\sum_{i} {N(t)}_{\begin{aligned} alive, \\ survived LC at time \leq t, \\ stage i, \\ screening \end{aligned}}\times U_{i} \right)-{Disutilities}_{screening, FP, indeterminate}$$

$${QALY}_{no-screening}=\sum_{t} \left[ {N(t)}_{\begin{aligned} alive. \\ no LC, \\ no-screening \end{aligned}}\times U_{b}+\sum_{i} \left( {N(t)}_{\begin{aligned} dead, \\ stage i, \\ no-screening \end{aligned}}\times{\tau_{i}\times U}_{i}+{N(t)}_{\begin{aligned} alive, \\ survived LC at time \leq t, \\ stage i, \\ no-screening \end{aligned}}\times U_{i} \right) \right]$$

$\frac{\Delta QALY}{N_{0}}=\frac{{QALY}_{screening}-{QALY}_{no-screening}}{N_{0}}=\frac{1}{N_{0}}\sum_{t} \left[ \sum_{i} \left( {N(t)}_{\begin{aligned} dead, \\ stage i, \\ screening \end{aligned}}-{N(t)}_{\begin{aligned} dead, \\ stage i, \\ no-screening \end{aligned}} \right)\times{\tau_{i}\times U}_{i}+\sum_{i} \left( {N(t)}_{\begin{aligned} survived LC at time \leq t, \\ stage i, \\ screening \end{aligned}}-{N(t)}_{\begin{aligned} survived LC at time \leq t, \\ stage i, \\ no-screening \end{aligned}} \right)\times U_{i}-{N\left( t \right)}_{\begin{aligned} alive. \\ overdiagnosed, \\ screening \end{aligned}}\times U_{b}--{Disutilities}_{screening, FP, indeterminate} \right]$,

where:

- ${N(t)}_{\begin{aligned} alive. \\ no LC, \\ screening \end{aligned}}$and ${N(t)}_{\begin{aligned} alive. \\ no LC, \\ no-screening \end{aligned}}$ are the cumulative number of people alive at time *t* in the screening and no- screening scenarios respectively, never diagnosed with lung cancer. Note that their difference gives the cumulative number of over-diagnosed cases in the screening scenario still alive at time *t*.
- ${N(t)}_{\begin{aligned} dead, \\ stage i, \\ screening \end{aligned}}$ and ${N(t)}_{\begin{aligned} dead, \\ stage i, \\ no-screening \end{aligned}}$are the number of cases diagnosed in year *t* in stage *i* who died in the screening and no-screening scenarios.
- ${N(t)}_{\begin{aligned} survived LC at time \leq t, \\ stage i, \\ screening \end{aligned}}$ and ${N(t)}_{\begin{aligned} survived LC at time \leq t, \\ stage i, \\ no-screening \end{aligned}}$ are the cumulative number of cases diagnosed in stage *i*, who survived LC and were still alive at time *t*, in the screening and no-screening scenarios.
- ${N(t)}_{\begin{aligned} alive, \\ overdiagnosed, \\ screening \end{aligned}}$is the cumulative number of overdiagnosed cases alive at time t.
- $\tau_{i}$ is the approximate mean survival time of stage *i* (for cases who died).
- $U_{b}$ is the baseline utility.
- $U_{i}$ is the utility weight for those with lung cancer diagnosed at stage *i*.
- ${Disutilities}_{screening, FP, indeterminate}$ are the disutilities applied for screening, false positives and indeterminate results defined as follows:
  - ${Disutilities}_{screening}=\sum_{t,screen} \left( {N(t)}_{\begin{aligned} alive. \\ no LC, \\ screening \end{aligned}}\times d_{s}\times2/52 \right)$
  - ${Disutilities}_{FP}=\sum_{t,screen} \left( {N(t)}_{\begin{aligned} false \\ positives \end{aligned}}\times\left( U_{b}-U_{I} \right)\times3/12 \right)$
  - ${Disutilities}_{indeterminate}=\sum_{t,screen} \left( {N(t)}_{\begin{aligned} indeterminate \\ results \end{aligned}}\times d_{ind}\times3/12 \right)$,

where the sum of the disutilities is over the screen time points, ${N(t)}_{\begin{aligned} alive. \\ no LC, \\ screening \end{aligned}}$was previously defined, ${N(t)}_{\begin{aligned} false \\ positives \end{aligned}}$ and ${N(t)}_{\begin{aligned} indeterminate \\ results \end{aligned}}$are the number of false positive cases and indeterminate results respectively found at time t, $d_{s}$ and $d_{ind}$ are the disutilities for screening and indeterminate results. The disutilities for screening are applied during two weeks (out of 52 in a year), and the disutilities for false positives and indeterminate results are applied for 3 months.

The main contribution to the difference in QALYs between the screening and no-screening scenario was generated mostly by the difference in cases who survived and to a lesser extent by the over-diagnosed cases. The difference arising from the mean survival times for cases who died was minor and contributed only a small correction to $\Delta QALY$.

The number of LC cases who survived and the number of LC deaths in each cancer stage were calculated based on the number of cases diagnosed, the stage distribution at diagnosis and the relative 5-survival by stage, age group and sex.

Explicitly, the number of deaths and the number of cases who survived in stage *i* at a given time *t*, following a LC diagnosis (in both the screening and no-screening scenarios were calculated as follows:

1. ${N(t)}_{\begin{aligned} LCdeaths, \\ stage i, \\ scenario \end{aligned}}={N(t)}_{\begin{aligned} LCdeaths, \\ scenario \end{aligned}}\times P\left( {stage i}/{LCdeath} \right)={N(t)}_{\begin{aligned} LCdeaths, \\ scenario \end{aligned}}\times\frac{P(stage i)\times P\left( LC{death}/{stage i} \right)}{P(LCdeath)}$
2. ${N(t)}_{\begin{aligned} survived, \\ stage i, \\ scenario \end{aligned}}={N(t)}_{\begin{aligned} survived, \\ scenario \end{aligned}}\times P\left( {stage i}/{survived} \right)={N(t)}_{\begin{aligned} survived, \\ scenario \end{aligned}}\times\frac{P(stage i)\times P\left( {survived}/{stage i} \right)}{P(survived)}$,

- $P\left( {stage i}/{LCdeath} \right)$ and $P\left( {stage i}/{survived} \right)$ stand for the probabilities of a LC diagnosis in stage *i* given that the case died or survived, respectively.
- $P(stage i)$ is the probability of stage *i* (given a LC diagnosis), and was obtained from the age specific stage distribution at diagnosis (or the shifted stage distribution for the screening scenario).
- $P\left( {survived}/{stage i} \right)$ is the probability of surviving LC given a diagnosis in stage *i*, and it was approximated as 5-year, age specific, conditional survival of stage *i*.
- $P\left( LC{death}/{stage i} \right)$ is the probability of a LC death given a diagnosis in stage *i* and it was equal to $1-P\left( {survived}/{stage i} \right)$.
- $P(survived)$ is the probability of surviving LC following a LC diagnosis in any stage and it was equal to $\sum_{i} P(stage i)\times P\left( {survived}/{stage i} \right)$.
- $P(LCdeath)$ is the probability of a LC death following a LC diagnosis in any stage and it was equal to $1-P(survived)$.
- ${N(t)}_{\begin{aligned} survived, \\ scenario \end{aligned}}$ was calculated as the difference between the cases diagnosed at time *t* and the LC deaths at time *t*, where the number of cases diagnosed was obtained from eq. 1.

The stage distribution at diagnosis was based on the AIHW data for LC incidence by stage, age group and sex. However, the 5-year survival data (by stage, age group and sex), available at the AIHW database was very limited. Since only partial data were available, the missing survival values were estimated by assuming decreasing linear functions for survival as a function of age for each stage, while minimising the sum of squared differences, estimated vs. actual data, of the overall 5-survival by stage (for each age group) and by age (for each stage).

The survival results obtained were assumed to be the same in both scenarios, except for the survival of stage I which was corrected in the screening scenario to account for over-diagnosed cases (we assumed that all over-diagnosed cases were stage I and that all of them survive LC).

Variations in the 5-year survival by age and stage were studied in sensitivity analyses.

# **One-Way Sensitivity Analyses and Probabilistic Sensitivity Analysis**

One-way sensitivity analyses were undertaken to assess the isolated parameter uncertainty. Details on each of the assumptions tested, the range of values for each parameter and the distributions assigned to them in the probabilistic sensitivity analysis (PSA) are given below:

**Mortality benefit**: One-way and probabilistic sensitivity analyses were conducted on (1) a range of values for the mortality benefit during the screening phase and on (2) its behaviour after the screening phase. We varied the value of the hazard ratio (defined as 1 minus the mortality benefit) by plus or minus 8% (NLST) and 15% (NELSON) of the base case value, and explored the extreme cases in which the mortality benefit drops immediately to zero after the last scan or remains constant for 3 years (NLST) or 4 years (NELSON). The resultant ranges of cumulative relative risk for LC mortality were 0.73 – 0.94 after 6 years of follow up (NLST) and 0.66-0.94 after 10 years (NESLON), consistent with the 95% confidence intervals reported in the trials^[21,22]^.

**LC and all-cause hazard ratios for eligible groups**: We varied the values of the hazard ratios between the 95% confidence limits. The all-cause hazard ratios (which has the opposite effect on the CER to the LC HR) were also varied between the 95% confidence limits but keeping the assumption that eligible (high risk) participants had the same all-cause HR as an Australian who currently smokes compared to those that never smoked. In the PSA, lognormal distributions were assumed for the hazard ratios.

**Costs**: One-way sensitivity analyses were conducted on a range of values for the (1) cost per screen ($200-$400); (2) cost of false positive follow-up (±20% of the base case value); (3) cost of treatment by for all stages (x0.5 – x2) of the base case value); this wide range was chosen to account for the high standard deviations in the costs of treatment reported in Goldsbury et.al.^[3]^ and additional uncertainties arising from changes in the costs during the years since 2016; (4) Cost of LC diagnosed at stage IV (x2, x4). Recent studies have shown that the total healthcare costs of stage IV has doubled since 2016 due to increased expenditure on targeted and immunotherapies. Multiplying the total healthcare cost of stage IV LC by a factor of 4 is an extreme case allowing for possible additional, future increments in this cost. (5) Pre-diagnosis costs in the screening scenario for cases detected during the screening phase (-80%). This reduction relaxes our conservative base case assumption, that pre-diagnosis costs are equal in both scenarios. It is reasonable to expect that screen-detected cases would not have pre-diagnosis costs related to managing undiagnosed, symptomatic disease in addition to the cost of screening. In the PSA we assigned normal distributions to the cost per screen and cost of false positives, and skew normal distributions for the cost per stage (independently for each stage) and for the cost pre-diagnosis. In addition, a threshold analysis was performed for the cost of an LDCT screen, in order to identify the cost for which the screening scenario is cost effective (assuming a willingness to pay threshold of AU$30,000 – AU$50,000 per QALY gained).

**False positive**, **follow-up CT rates and over-diagnosis:** We varied the values of the false positive, follow-up CT and overdiagnosis rates between the lower and upper limits of the 95% confidence intervals, obtained by assuming a beta distribution and applying the numbers reported in the trials. The magnitude of over-diagnosis as estimated from LDCT screening trials depends critically on the length of the follow-up after the final screen. The overdiagnosis rate reported in the NELSON trial (8.9% out of the screen detected cases or 5.25% out of all the cases in the LDCT arm) can be considered the upper limit of overdiagnosis, since an appropriate estimation of the level of over-diagnosis would require additional years of follow up^[21]^. The extended follow-up data from the NLST showed that there was no significant difference in LC incidence between the LDCT and control groups after 11 years of follow-up^[22]^ (their updated estimate for the overdiagnosis rate was 3% out of the screen detected cases, or 1.84% out of all the cases in the LDCT arm). However, to maintain a conservative approach, we varied the over-diagnosis rates between the upper and lower limits of the 95% confidence interval of each trial (NELSON: 3%-8%, NLST: 1.12% - 2.8%). Beta distributions were assumed for these three parameters in the PSA.

**Time Horizon:** A one-way sensitivity analysis studied 20-year and 10-year time horizons as compared with the lifetime horizon used in the base case. Variations in the time horizon were not included in the PSA.

**Stage shift and stage “unknown” assumption:** The stage shift factors were varied between the lower and upper limit of their 95% confidence intervals, obtained by assuming beta distributions for the number of cases “transitioning” from each stage to stage I. The same distributions were assigned in the PSA. In addition, the extreme assumptions of all the “unknown” cases being stage IV or stage I were explored. In the PSA, the “unknown” option was varied discretely among 4 possibilities of equal probability.

**5-year survival inputs:** The survival inputs were varied between the limits which reproduce the 95% confidence interval of the incidence as explained previously. In the PSA the survival input was varied discretely among four possible values.

**Utility weights:** All utility weights were varied between the lower and upper limits of the 95% confidence intervals. Uncertainty around utility data used Beta distributions. The parameters were set so that the 95% central interval of the resulting distribution matched the 95% confidence interval from the published literature. The disutilities associated with screening itself and with follow up scans were varied from 0 to twice the baseline values. Normal distributions were assigned to these disutilities in our PSA. The false positive disuitlity in our base case assumed the worst case scenario in which false positives had the same utility as stage I lung cancer. The effect of setting this parameter to zero was studied in our one-way sensitivity analysis, and a skew distribution was assumed in the PSA. In addition, we evaluated the impact of assuming that LC cases diagnosed at stage I return to the baseline utility value after 1 year (this effect was not included in the PSA).

The combined parameter uncertainty throughout the model was evaluated with a PSA which simulated 10,000 possible ICER estimates by taking random values of the parameters from each of the parameter distributions (each independently).

Table s5 summarizes the parameters included in the PSA along with the probability distributions assigned to each one of them.

**Table s5** Summary of the parameters included in the PSA along with the probability distributions assigned to each parameter**.**

| **Parameter** | **Distribution** | **Plots** | |
| --- | --- | --- | --- |
| Mortality hazard ratio during the screening phase (1 – mortality benefit). | Normal, centered at the base case values, with std deviations of 7.5% (NELSON) and 4% (NLST) (rejecting values lower than zero or greater than 1). | 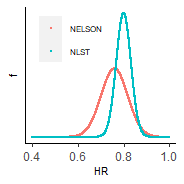 | |
| Time to apply benefit after trial ends | Uniform, discrete values.  NLST:0,1,2,3  NELSON:0,1,2,3,4 |  | |
| LC mortality HR for eligible individuals by smoking status | **Lognormal.**  Parameters set so match the reported 95% CI. | 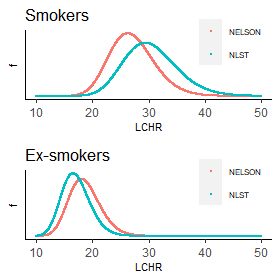 | |
| Overdiagnosis | **Beta distributions.**  a = Estimated number of overdiagnosed cases as reported in the trials (extended follow up for NLST).  b = Total number of LC cases in the screening scenario - a. | 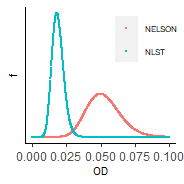 | |
| False Positives | **Beta distributions.**  a = Number of FP as reported in the trials.  b = Total number of screens - a. | 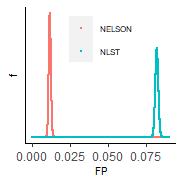 | |
| Indeterminate Results | **Beta distributions.**  a = Number of indeterminate results as reported in the trials (false positives requiring only further imaging in the NLST).  b = Total number of screens - a. | 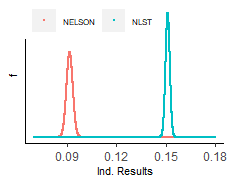 | |
| Stage Shift | **Beta distributions.**  a_i_= Estimated number of cases transitioning from stage i to stage 1.  This estimate is given by $n_{i}f_{i}$ as detailed in equation 6 (in “Detailed assumptions regarding stage shift and stage “unknown.”), using the numbers provided in table s4.  b_i_ = Total number of cases in stage i in the non-screening scenario – a_i_  (i = II, III or IV) | 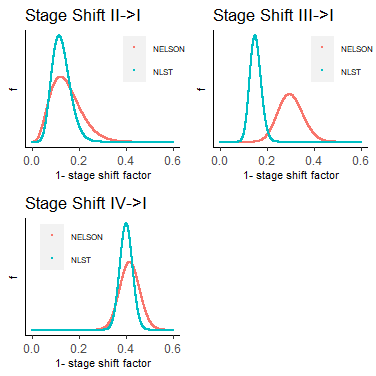 | |
| Stage “unknown” composition | **Uniform, discrete values.**  1: All “unknowns” are stage I  2: “unknowns” are redistributed according to the original distribution from stage I to IV, renormalized to 1.  3: Base case (60% stage IV, 21% stage III, 12% stage II and 7% stage I)  4: All “unknowns” are stage IV. | | |
| All Cause mortality HR by smoking status (by age group and sex) | **Lognormal.**  Mean values and SE obtained from Wade S. et. al. ^[19]^.  The ln(HR) for each age group and sex were calculated according to  $ln(HR)=\ln\left( <HR> \right)+p*SElnHR$, where $<HR>$ and $SElnHR$ are the mean value and standard error corresponding to the given age group and sex, and *p* is a random number drawn from a standard normal distribution.  The same hazard ratios were assigned to individuals who had quit smoking. | | |
| Survival Input | **Uniform, discrete values.**  1: 5-year survival from data (AIHW)  2: Calculated values which reproduce the incidence from data.  3: 0 (incidence rate = mortality rate)  4: Calculated values which reproduce the upper limit of the incidence from data. | | |
| Baseline utilities | **Beta distributions.**  The parameters were set to match the 95% CI available in the data. | | 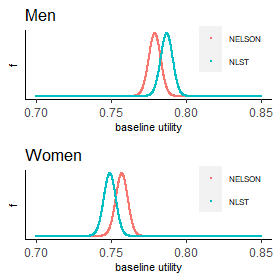 |
| LC utilities by stage. | **Beta distributions.**  The standard errors used for calculating the 95% CI of the mean value were estimated from the reported standard deviations and sample size. | | 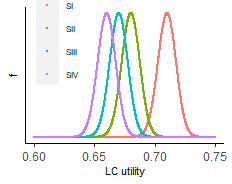 |
| Indeterminate results and screening disutilities. | **Normal distribution**  Centered at the base case (0.02) with standard deviation 0.01 (and rejecting values greater than 1 or lower than zero) | | 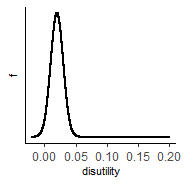 |
| False positive disutility | **Skew normal distribution**  The disutility applied to false positives is given by:  Dis. FP = x*(U_baseline_– U_LC StageI_),  where x is a random number (x=1 in our base case, which is the worst-case scenario).  The parameters of the distribution were set so that x lies between 0.3 and 1 in 95% of the cases. | | 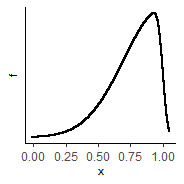 |
| Cost of LDCT scan. | **Normal Distribution**  Mean = AU$300 (base case)  Std dev = AU$50  (In 95 % of the cases the cost lies between AU$202 and AU$ 398).  Values lower than zero were rejected. | | 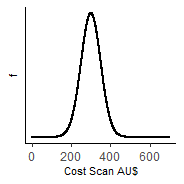 |
| Cost of treatment by stage. | **Skew Normal Distributions**  The parameters were set so that the 95% central interval of the resulting distribution matched the range (x0.5 – x2) of the base case values | | 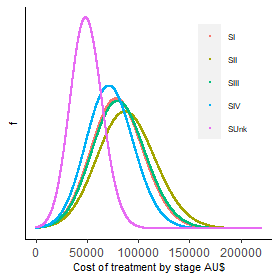 |
| Cost of false positive follow up. | **Normal Distribution**  mean = AU$861.8  std dev = AU$ 90  (In 95 % of the cases the cost was between -20% to +20% of the base case value) | | 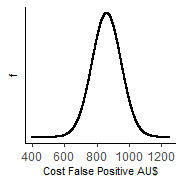 |
| Cost pre-diagnosis in the screening scenario during the screening phase. | **Skew Normal Distribution**  The factor by which the cost pre-diagnosis was reduced in the screening scenario varied from 0 (no reduction at all) to 1, following a skewed distribution so that in 95% of the cases a value under 0.8 was obtained.  Negative values and values greater than one were rejected. | | 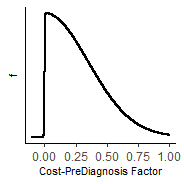 |

# **References**

1. R Core Team (2020). R: A language and environment for statistical computing. R Foundation for Statistical Computing, Vienna, Austria. URL <https://www.R-project.org/>.
2. RStudio Team (2020). RStudio: Integrated Development Environment for R. RStudio, PBC, Boston, MA URL http://www.rstudio.com/
3. Goldsbury DE, Weber MF, Yap S, Rankin NM, Ngo P, Veerman L, et al. Health services costs for lung cancer care in Australia: Estimates from the 45 and Up Study. *PLoS One*. 2020;15(8): e0238018.
4. Marshall HM, Finn N, Bowman RV, Passmore LH, McCaul EM, Yang IA, et al. Cost of screening for lung cancer in Australia. *Intern Med J* 2019;49(11):1392–9.
5. Wickham H, Averick M, Bryan J, Chang W, McGowan LD, [François](https://joss.theoj.org/papers/by/Romain%20Fran%C3%A7ois) R, et al., (2019). Welcome to the tidyverse. Journal of Open Source Software, 4(43), 1686, <https://doi.org/10.21105/joss.01686>
6. Wickham H (2011). The Split-Apply-Combine Strategy for Data Analysis. Journal of Statistical Software, 40(1), 1-29. URL <http://www.jstatsoft.org/v40/i01/>
7. Wickham H. ggplot2: Elegant Graphics for Data Analysis. Springer-Verlag New York, 2016.
8. Baptiste Auguie (2017). gridExtra: Miscellaneous Functions for "Grid" Graphics. R package version 2.3. <https://CRAN.R-project.org/package=gridExtra>.
9. Azzalini, A. (2021). The R package 'sn': The Skew-Normal and Related Distributions such as the Skew-t and the SUN (version 2.0.0). URL <http://azzalini.stat.unipd.it/SN/>.
10. Millard SP (2013). _EnvStats: An R Package for Environmental Statistics_. Springer, New York. ISBN 978-1-4614-8455-4, URL: https://www.springer.com
11. Australian Institute of Health and Welfare (AIHW Australian Cancer Database 2017).
12. 45 and Up Study Collaborators, Banks E, Redman S, Jorm L, Armstrong B, Bauman A, Beard J, Beral V, Byles J, Corbett S, Cumming R, Harris M, Sitas F, Smith W, Taylor L, Wutzke S, Lujic S. Cohort profile: the 45 and up study. Int J Epidemiol. 2008 Oct;37(5):941-7.
13. Weber M, Yap S, Goldsbury D, Manners D, Tammemagi M, Marshall H, et al. Identifying high risk individuals for targeted lung cancer screening: Independent validation of the PLCOm2012 risk prediction tool. Int J Cancer. 2017 Jul 15;141(2):242-253. doi: 10.1002/ijc.30673.
14. Jaro MA. Probabilistic linkage of large public health data files. Stat Med. 1995 Mar 15-Apr 15;14(5-7):491-8. doi: 10.1002/sim.4780140510. PMID: 7792443.
15. Kelman CW, Bass AJ, Holman CD. Research use of linked health data--a best practice protocol. Aust N Z J Public Health. 2002;26(3):251-5. doi: 10.1111/j.1467-842x.2002.tb00682.x. PMID: 12141621.
16. Bentley JP, Ford JB, Taylor LK, Irvine KA, Roberts CL. Investigating linkage rates among probabilistically linked birth and hospitalization records. BMC Med Res Methodol. 2012 Sep 25;12:149. doi: 10.1186/1471-2288-12-149. PMID: 23009079; PMCID: PMC3533905.
17. Weber MF, Sarich PE, Vaneckova P, Wade S, Egger S, Ngo P, et al. Cancer incidence and cancer death in relation to tobacco smoking in a population-based Australian cohort study. Int J Cancer. 2021 May 20. doi: 10.1002/ijc.33685.
18. Australian Bureau of Statistics. *Population Projections, Australia, 2017 (Base) - 2066*. ABS website; 2018. <https://www.abs.gov.au/statistics/people/population/population-projections-australia/2017-base-2066>.
19. Wade S, Weber MF, Sarich P, Vaneckova P, Behar-Harpaz S, Ngo PJ, et al. Bayesian calibration of simulation models: A tutorial and an Australian smoking behaviour model. arXiv preprint arXiv:220202923. 2022.
20. Aberle DR, Adams AM, Berg CD, Black WC, Clapp JD, Fagerstrom RM, et al. Reduced lung-cancer mortality with low-dose computed tomographic screening. *N Engl J Med.* 2011, 365 (5): 395-409.
21. de Koning HJ, van der Aalst CM, de Jong PA, Scholten ET, Nackaerts K, Heuvelmans MA, et al. Reduced Lung-Cancer Mortality with Volume CT Screening in a Randomized Trial. *N Engl J Med.* 2020, 382 (6): 503-513.
22. National Lung Screening Trial Research Team. Lung Cancer Incidence and Mortality with Extended Follow-up in the National Lung Screening Trial. *J Thorac Oncol*. 2019;14(10):1732-1742.
23. Ngo PJ, Wade S, Vaneckova P, Behar-Harpaz S, Caruana M, Cressman S, et al. Health utilities for participants in a population-based sample who meet eligibility criteria for lung cancer screening. Lung Cancer. 2022;169:47-54.
24. Ngo PJ, Wade S, Banks E, Karikios DJ, Canfell K, Weber MF. Large-Scale Population-Based Surveys Linked to Administrative Health Databases as a Source of Data on Health Utilities in Australia. Value Health. 2022: S1098-3015(22)00195-4.
25. Tramontano AC, Schrag DL, Malin JK, Miller MC, Weeks JC, Swan JS, et al. Catalog and comparison of societal preferences (utilities) for lung cancer health states: results from the Cancer Care Outcomes Research and Surveillance (CanCORS) study. *Med Decis Making*. 2015;35(3):371-387.
